# Assortative mate preferences for height across short-term and long-term relationship contexts in a cross-cultural sample

**DOI:** 10.3389/fpsyg.2022.937146

**Published:** 2022-08-25

**Authors:** Katarzyna Pisanski, Maydel Fernandez-Alonso, Nadir Díaz-Simón, Anna Oleszkiewicz, Adrian Sardinas, Robert Pellegrino, Nancy Estevez, Emanuel C. Mora, Curtis R. Luckett, David R. Feinberg

**Affiliations:** ^1^Centre National de la Recherche Scientifique (CNRS), Laboratoire Dynamique du Language, Université Lyon 2, Lyon, France; ^2^Institute of Psychology, University of Wrocław, Wrocław, Poland; ^3^Max Planck Institute for Biological Cybernetics, Tübingen, Germany; ^4^Interdisciplinary Center on Cognition for Teaching and Learning, University of the Republic, Montevideo, Uruguay; ^5^Smell and Taste Clinic, Department of Otorhinolaryngology, Technische Universität Dresden, Dresden, Germany; ^6^Department of Psychology, University of Western Ontario, London, ON, Canada; ^7^Center for Sensory Science, University of Tennessee, Knoxville, TN, United States; ^8^Monell Chemical Senses Center, University City Science Center, Philadelphia, PA, United States; ^9^Neurodevelopment Department, Cuban Neurosciences Center, Havana, Cuba; ^10^Department of Animal and Human Biology, University of Havana, Havana, Cuba; ^11^Department of Psychology, Neuroscience and Behaviour, McMaster University, Hamilton, ON, Canada

**Keywords:** assortative mating, body size, relationship context, relationship length, cross-cultural, mate choice, mate preferences, sexual selection

## Abstract

Height preferences reflecting positive assortative mating for height—wherein an individual’s own height positively predicts the preferred height of their mate—have been observed in several distinct human populations and are thought to increase reproductive fitness. However, the extent to which assortative preferences for height differ strategically for short-term versus long-term relationship partners, as they do for numerous other indices of mate quality, remains unclear. We explore this possibility in a large representative sample of over 500 men and women aged 15–77 from Canada, Cuba, Norway and the United States. Participants’ own heights were measured, and they indicated their height preferences for a long-term and short-term mate using graphic stimuli containing metric indices. Replicating the “male-taller norm,” participants on average preferred taller-than-average male mates, and shorter-than-average female mates. Positive assortative preferences for height were observed across sexes and samples, however the strength of these height preferences varied with relationship context for men, and not for women. Taller men preferred relatively shorter women for short-term relationships than for long-term relationships, indicating stronger assortative preferences for height in a long-term context. These results provide preliminary evidence that, in addition to mate preferences for other physical traits related to mate quality such as masculinity in the body, face, and voice, assortative preferences for height do vary as a function of expected relationship length, but this was surprisingly only observed in preferences for female height.

## Introduction

One hundred and fifty years ago, Darwin published his now remarkably influential theory on sexual selection, emphasizing the role of phenotypic qualities in mate choice decisions (Darwin, 1871). Since then, a vast body of literature offers converging evidence that mate choice decisions are critical for the reproductive success of humans and all other sexually reproducing organisms, and as such mate preferences have been largely shaped by sexual selection to maximize fitness (Andersson, 1994; Conroy-Beam et al., 2019a). Mate preferences in humans are intensively studied and shown to range from preferences for specific character or personality traits, such as kindness and honesty, to preferences for physical traits, such as age or physical attractiveness, that ostensibly function to target mates of high genetic quality, resource holding potential, and/or reproductive potential (Shackelford et al., 2005; Roberts and Little, 2008; Walter et al., 2020).

Mate preferences for physical height in humans are observed in both sexes and across diverse cultures (Courtiol et al., 2010; Pisanski and Feinberg, 2013; Yancey and Emerson, 2014; Stulp et al., 2017). This is perhaps not surprising given that height predicts a range of life-history and fitness-related factors including health, socioeconomic status, dominance, and reproductive maturity (Pisanski and Feinberg, 2013; Stulp and Barrett, 2016 for reviews). Body size in humans, like in many other animals (Andersson, 1994), is a sexually dimorphic trait under natural and sexual selection that is both highly heritable and susceptible to environmental influences. For instance, there is evidence that taller men and women have, on average, a lower risk of mortality than do relatively shorter individuals (Wormser et al., 2012). Shorter-than-average men in turn exhibit the lowest rates of both social and reproductive success (Stulp and Barrett, 2016), particularly in industrialized or western populations where stature predicts substantial variance in social status (Judge and Cable, 2004). Among women, a basic principle of allocation underscores a trade-off between skeletal growth and reproduction, with shorter women typically reaching sexual menarche, and thus reproductive maturity, sooner than taller women, and as a consequence giving birth to their first child relatively earlier (Stearns, 2000; Stulp and Barrett, 2016). At the same time, short mothers experience more complications during pregnancy and parturition (in part due to their small pelvis size) and higher rates of child morbidity and mortality in both developing and industrialized countries (Stearns, 2000; Stulp and Barrett, 2016).

The mate height preferences of both sexes are likely to reflect adaptive mate choice decisions (Kuijper et al., 2012; Buss and Schmitt, 2019), however it remains unclear exactly which aspects of mate quality that are linked to height can explain the most variance in height preferences (Kowal et al., 2021). Height preferences have been studied using a range of methodologies including numerical self-report, judgments of figure drawings, and mate-choice relevant tasks (e.g., outcomes of speed dates or dating advertisements; see Courtiol et al., 2010 for review). These studies offer converging evidence for a “male-taller-norm” among young heterosexual western adults: women generally indicate preferences for men taller than themselves, whereas men indicate preferences for relatively shorter women (for reviews, see Pisanski and Feinberg, 2013; Stulp and Barrett, 2016). A similar pattern emerges in actual mated pairs at a rate much higher than expected by chance (Gillis and Avis, 1980; Stulp et al., 2013b). Western women generally prefer men who are taller than the average man in their given population, for example, men that are around 180 cm tall (Beigel, 1954; Salska et al., 2008; Courtiol et al., 2010). However, women’s preferences for above-average height in men appear to asymptote within one or two standard deviations of the average (Beigel, 1954; Pawlowski, 2003; Fink et al., 2007; Salska et al., 2008; Courtiol et al., 2010; Stulp et al., 2013a, 2013b; i.e., the “male-not-too-tall norm”). This aligns with evidence that, although taller men are generally healthier than average-height or short men, very tall men have relatively higher rates of health complications (Stulp et al., 2014; Stulp and Barrett, 2016).

Men’s preferences for women’s heights have consistently been shown to be weaker, less consistent, and less robust than women’s preferences for men’s heights (Pierce, 1996; Salska et al., 2008; Courtiol et al., 2010; Stulp et al., 2013a). This may reflect evolved and/or sociocultural sex differences in mate preferences (Walter et al., 2020), wherein, for example, body size in men can index dominance and resource holding potential—traits that are more likely to have been sexually selected among men than women. Among women, indices of youth and fertility are more likely to have been selected for, and indeed are reflected in the general mate preferences of men (Buss and Schmitt, 1993; Shackelford et al., 2005; Walter et al., 2020). Nevertheless, female height can still index health and fecundity to potential mates. The limited existing research on preferences for women’s heights suggests that western heterosexual men prefer average or shorter-than-average women, however others report the opposite or no preference at all (Courtiol et al., 2010 for review). Studies examining the preferences of men who identify as non-heterosexual also show mixed findings, with support both for and against a general tendency to prefer a partner taller than oneself (Valentova et al., 2014, 2016). In addition, such preferences appear to be moderated by a number of factors, including the height of the rater (Valentova et al., 2014).

Indeed, an individual’s own height is known to predict their preferred height in a mate. Beyond absolute height preferences, several studies have found evidence for positive assortative preferences for height, such that taller individuals tend to prefer taller mates and vice versa (Pawlowski, 2003; Kurzban and Weeden, 2005; Fink et al., 2007; Courtiol et al., 2010; Stulp et al., 2013b). Assortative mating in humans and other animals is most often positive, that is, occurs between two individuals that share a given phenotypical quality (Thiessen and Gregg, 1980; Conroy-Beam et al., 2019b). Such a preference can be adaptive if it increases genetic homology while avoiding inbreeding (Thiessen and Gregg, 1980). Researchers have posited that positive assortative preferences for height may increase the likelihood that height differences between mated pairs are not extreme. Indeed, preferences for relative height in humans typically do not exceed a difference of 25 cm between heterosexual mates (Pawlowski, 2003; Fink et al., 2007; Salska et al., 2008). Positive assortative preferences for height may thus function to increase one’s pool of potential partners (Pawlowski, 2003) or the quality of resultant offspring. Height is highly hereditable (*h* = 0.80; Stulp and Barrett, 2016), hence the offspring of two tall individuals will likely be tall as well. There is also some evidence that extreme height differences between mated pairs predict a higher likelihood of birth complications (Stulp et al., 2011).

While mate preferences can differ somewhat from actual mate choices due to conflicts of interest between the sexes and numerous factors that limit the extent to which certain individuals can attain a preferred or high-quality mate (Jiang et al., 2013; Stulp et al., 2013b), a recent meta-analysis indicates weak positive assortative mating for height in heterosexual couples across 43 western (*r* = 0.25) and non-western (*r* = 0.21) countries (Stulp et al., 2017), with no cross-cultural differences (Stulp et al., 2017). Notably, assortative preferences for height have not been consistently replicated, for example, in non-traditional societies such as several tribes on the African continent including the Hadza, Himba, Datoga and Tsimane’ (Sear and Marlowe, 2009; Sorokowski et al., 2011, 2015; Sorokowski and Butovskaya, 2012).

The present study tests whether preferences for mate height differ for committed long-term compared to uncommitted short-term relationships in a large cross-cultural sample of adults. While most human societies practice marriage, short-term liaisons among single people or through serial monogamy and extra-pair affairs are even more common in our species (Buss and Schmitt, 1993 for review). Many studies have shown that mate preferences for traits linked to mate quality can differ for short- versus long-term relationships (Buss and Schmitt, 1993, 2019). Much of this research has focused on female preferences for male androgen-mediated traits, such as facial, vocal and bodily masculinity. In general, women show relatively stronger preferences for androgen-mediated male traits in the context of a short-term versus long-term relationship (Little et al., 2011), presumably because the potential benefits of choosing a male mate with relatively high androgen levels (e.g., higher immunocompetence that may be passed to offspring) outweigh the potential costs (e.g., higher risk of infidelity and decreased investment) in a short-term but not long-term relationship (Puts et al., 2012 for review; e.g., Buss and Schmitt, 1993, 2019). However, mate preferences have also been shown to vary by relationship context for traits that may not be directly linked to androgen-levels, but that nevertheless predict characteristics relevant in a potential long-term versus short-term mate. This may include, for example, relatively stronger preferences for intelligence and honesty in long-term mates, and for sex drive or athleticism in short-term mates (Buss et al., 1990; Regan et al., 2000; Muggleton and Fincher, 2017).

Despite being a sexually dimorphic trait in humans, height does not appear strongly and consistently related to circulating testosterone levels in adult men (Kowal et al., 2021). Thus, while female preferences for male height may not vary by relationship context as a function of variable androgen-linked immunity benefits *per se*, as they do for androgen-mediated traits, other benefits could be gained from such a preference when expressed by either sex. Indeed, this is because height is linked more broadly to mate quality and health benefits in both sexes, and to fecundity in women. To our knowledge, only one previous study examined height preferences for short-term versus long-term mates, and that study focused only on heterosexual women’s preferences for sexual dimorphism in hypothetical mate pairs. Testing a sample of nearly 150 Polish women, Pawlowski and Jasienska (2005) found that women preferred relatively taller male mates in a short-term than long-term mating context. However, the effect of relationship length was small, as more than half of the women showed the same height preference regardless of relationship context (Pawlowski and Jasienska, 2005). No previous study has tested whether preferences for women’s heights differ by relationship context.

Here, we test the prediction that positive assortative preferences for height will be observed across four human populations and further explore whether these preferences differ by hypothetical relationship context for both men’s and women’s heights. This exploratory study included a diverse sample of over 500 male and female raters aged 15 to 77. To address the overabundance of studies on the height preferences of predominantly western undergraduate students (Courtiol et al., 2010 for review; Pisanski and Feinberg, 2013), participants were recruited from both rural and urban regions in Canada, Cuba, Norway and the United States. While some researchers have suggested that population-level height differences may influence height preferences (Pawlowski, 2003; Stulp and Barrett, 2016), evidence is lacking for cross-cultural differences in assortative mating for height (Pawlowski, 2003; Fink et al., 2007; Stulp et al., 2017). Indeed, if height preferences reflect long-standing evolved mechanisms in our species, they are likely to be relatively stable across cultures (Shackelford et al., 2005). Moreover, differences in height among same-sex individuals *within* each of the sampled countries (up to 50 cm) far outweighed the average differences in height between countries (Cubans were on average 6 cm shorter than same-sexed Norwegians, with North Americans falling in between these two extremes). We thus had no *a priori* predictions that country would explain significant variance in assortative preferences for mate height above and beyond individual differences in height. As a result, we did not test for differences between countries, but instead modeled the variation in height preferences across these countries, using country as a random-intercept term. Our analyses hence focus on testing for an effect of relationship context on assortative preferences for height in both men and women.

## Materials and methods

### Participants

Descriptive statistics for the ages, heights, and weights of men and women in each country are given in Table 1. Five-hundred and thirty-six participants took part (333 women, 203 men), aged 15–77 (mean age 25.8 ± 11.4 years). Participants were recruited from the general population (rural and urban) and from local universities in four countries: Canada (*n* = 143), Cuba (*n* = 187), Norway (*n* = 95) and the United States (*n* = 111), using a combination of recruitment methods ranging from online advertisements to word-of-mouth (self-reported nationalities are given in Supplementary Table S1).

**Table 1:** Descriptive statistics for age, height, and weight of each sample by sex and country.

Sex of rater	Country		*N*	Mean	SD	Min	Max
Men	All	Age	203	26	11.3	15.0	75.0
		Height (cm)		178.3	7.8	152.4	200.7
		Weight (kg)		76.5	16.9	47.8	149.7
	Canada	Age	46	18.5	1.7	17.0	28.0
		Height (cm)		179.2	7.2	160.1	190.5
		Weight (kg)		71.4	12.2	47.8	99.8
	Cuba	Age	86	23.1	3.2	19.0	32.0
		Height (cm)		176.0	6.9	160.0	190.0
		Weight (kg)		69.9	11.6	50.0	110.0
	Norway	Age	42	33.8	14.8	15.0	75.0
		Height (cm)		181.2	7.2	165.0	200.0
		Weight (kg)		85.6	18.1	54.0	125.0
	United States	Age	29	35.4	16.4	19.0	70.0
		Height (cm)		179.8	10.0	152.4	200.7
		Weight (kg)		90	21	61.2	149.7
Women	All	Age	333	25.7	11.4	15.0	77.0
		Height (cm)		164.9	7.5	130.0	190.5
		Weight (kg)		62.9	15.4	28.6	149.7
	Canada	Age	97	19.1	2.3	17.0	29.0
		Height (cm)		165.1	7.9	149.9	190.5
		Weight (kg)		60.6	12	38.6	98.9
	Cuba	Age	101	21.2	3.4	18.0	32.0
		Height (cm)		163	7.7	130.0	180.0
		Weight (kg)		56.4	8.2	40.0	84.0
	Norway	Age	53	28.9	13.5	15.0	67.0
		Height (cm)		167.6	5.7	154.0	177.0
		Weight (kg)		63.8	12.1	46.0	100.0
	United States	Age	82	37.1	13.5	20.0	77.0
		Height (cm)		165	7.3	144.8	188.0
		Weight (kg)		72.9	21.2	28.6	149.7

Overall, 93.3% of participants reported the opposite-sex as their preferred sex for a romantic relationship, 3.4% reported a preference for the same-sex, 2.8% for either sex, and 0.6% did not report their sexual orientation. While height preferences can differ between heterosexual and non-heterosexual samples (Valentova et al., 2014, 2016), our sample size of non-heterosexual participants was not large enough to test for group differences. Thus, all participants regardless of their sexual orientation or age were included in the statistical analyses reported in this paper. However, models including only heterosexual participants who self-reported as preferring the opposite-sex, and models including only participants aged 17 to 40 (i.e., spanning the most reproductively relevant years of the human lifespan), can be found in the Supplementary Materials. Excluding participants on the basis of their sexual orientation and/or age from analyses did not change any significance levels. Participants provided written informed consent. Depending on the country sample, they received course credit for taking part (Canada, United States), or a small gift (Cuba, Norway). The study was conducted according to the Declaration of Helsinki on Biomedical Studies Involving Human Subjects.

### Stimuli

**Figure 1:**
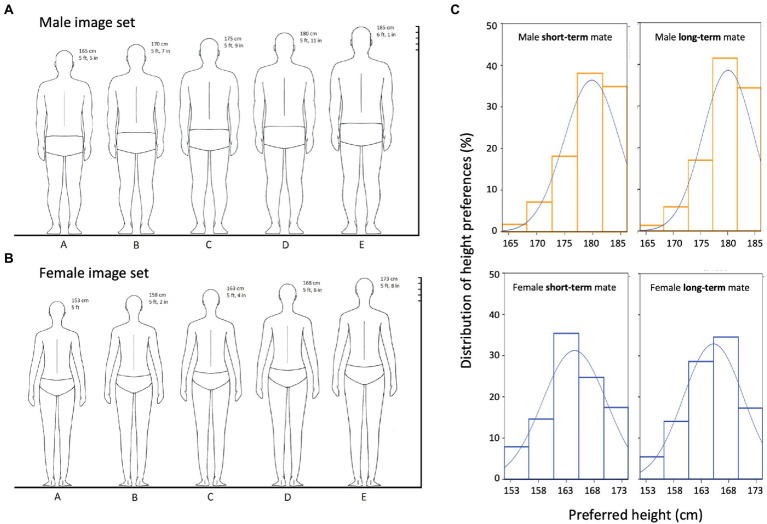
**(A,B)** Stimulus image set featuring back-facing silhouettes of men (panel **A**) and women (panel **B**) used to measure mate height preferences. See *Stimuli* for detailed descriptions of stimulus creation and parameters. **(C)** Distributions of raw absolute height preferences for male heights (top row) and female heights (bottom row) in short-term versus long-term relationship contexts.

Minimalistic graphic representations of back-facing male (Figure 1A) and female (Figure 1B) bodies were first generated by hand, scanned, and then digitally edited using Adobe Photoshop CS6. We controlled for body symmetry by inverse mirroring the left side of the body to the right (following Courtiol et al., 2010). In addition, aspect ratios between body width and height, and between the hips, shoulders and waist, were standardized across the scale. Bodies were positioned from shortest to tallest and labeled A to E, with heights given in both metric (cm) and imperial (feet, inches) units. The central figure marked “C” on the graphic represents average height for each sex, with adjacent bodies representing ±5 cm and ± 10 cm deviations from the mean for a range of 20 cm. Scale values derive from population statistics obtained for North American adults (Shields et al., 2008; NCD Risk Factor Collaboration, 2016), as this approximates height distributions for Central and Northern global populations (NCD Risk Factor Collaboration, 2016). Height scale values were selected so as to avoid extremes at either end of the average distribution of heights, and the same standardized scale was used for all participants in all four countries to allow for direct cross-cultural comparisons. Adding numerical labels representing absolute heights to our scale, rather than using a wholly visual representation of relative height differences between hypothetical pairs such as the sexual dimorphism in stature (SDS) scale (Pawlowski, 2003; Fink et al., 2007; Sorokowski et al., 2011), allowed for more precise numerical calculation of absolute height preferences and of the strength of the relationship between own and preferred height, in real units.

### Procedure

Participants were simultaneously presented with the graphic representations of men and women (Figures 1A,B), on two separate laminated pages of A4 paper (Canada, Cuba, Norway) or as two digital images scaled to the same size (United States). They were then asked to indicate their height preference for a mate of their preferred sex in the context of a stable long-term relationship (for example, marriage) and a short-term relationship (for example, a one-night stand) following previous work (Little et al., 2002; Feinberg et al., 2012; O’Connor et al., 2014). The order in which long-term or short-term preferences were tested was randomized. Participants were instructed that they could select more than one preferred height only if they strongly felt that they equally prefer multiple height options. However, multiple preferred heights were selected on only 2.6% of trials (28 of 1,072) and were thus averaged together to obtain a single preferred height value. Participants also reported their sex, age, and sexual orientation. Canadian, Cuban, and Norwegian samples (80% of participants) completed the study using pen and paper; their heights and weights were measured using metric tape and a digital scale. American participants completed the study online using survey software (Research Core, 2017, Qualtrics, Provo, Utah, United States); their heights and weights were self-reported.

## Models and results

For full statistical models, data, R code and output, and research stimuli see Supplementary Materials and online materials (Open Science Framework, https://osf.io/ah97w/).

We coded the response variable (Height Preference) in centimeters. Figure 1C shows the distribution of absolute height preferences for female and male mates in either relationship context. Table 2 reports absolute height preferences across countries. These absolute preference values (and their general distributions; Figure 1C) are notably based on rater’s raw responses and are largely given here for comparisons with previous studies. The values do not account for variance tied to individual raters or groups, including variance as a function of the rater’s own height, as we explore in our linear mixed effects models.

**Table 2:** Absolute (raw) height preferences by sex, country, and relationship context. These raw values do not account for variance due to individual or sample differences.

	Country	Relationship context	Preferred height (cm)	
			Mean	SEM
Preferred Heights for Women	Canada	Long-term	167.178	0.8792
		Short-term	165.565	0.9177
	Cuba	Long-term	164.802	0.6294
		Short-term	164.018	0.6824
	Norway	Long-term	165.976	0.7536
		Short-term	166.077	0.8289
	United States	Long-term	165.552	1.2353
		Short-term	164.517	1.3230
Preferred Heights for Men	Canada	Long-term	180.402	0.5046
		Short-term	179.629	0.5550
	Cuba	Long-term	179.208	0.4532
		Short-term	178.943	0.4470
	Norway	Long-term	179.971	0.7674
		Short-term	180.181	0.7814
	United States	Long-term	179.691	0.6629
		Short-term	179.962	0.6979

SEM = standard error of the mean.

To test our key hypothesis regarding assortative preferences for height by relationship context, we ran a series of linear mixed effects models using the R package lmerTest (Kuznetsova et al., 2017). In all models, we set REML to false, and fit with Maximum Likelihood to facilitate model comparison. We centered the response variable (Height Preference) on the sample mean (such that the intercept effects approximate a *t*-test, and the intercept itself is not 0 cm, an impossible value) and z-scored the Own Height variable. We then created models specifying random slopes and intercepts maximally with correlations between random effects and binary variables sum-to-zero coded (−0,5, 0.5) to reduce false positives and to create a full-factorial ANCOVA-like analysis (Barr, 2013). We entered all fixed-effects as random slopes at each participant’s random intercept (i.e., all main effects and all two-way and three-way interactions among own height, sex, and relationship context). When grouping data by self-reported free-response nationality or by country, we nested each participant within their nationality or country. We also fit random intercepts for age as a control. Because we had no formal predictions regarding the age or country/nationality of raters, we did not enter these as a fixed-effects terms and the factors are instead treated as nuisance variables.

Twenty-two participants chose not to indicate a height preference for either a long-term or short-term relationship partner, and their data were thus excluded from these models. For analyses we thus included the short-term and long-term preferences of 514 participants, totaling 1,028 observations, however this varies slightly depending on the analysis as some participants did not complete all demographic questions. The number of observations per analysis are noted in the Supplementary Materials in each model’s output.

The R code for Model 1 nesting participants by country is:

lmer(height_preference ~ height_z_scored × Sex × Relationship_context + (1 + height_z_scored × Sex × Relationship_context || ID_NUMBER:Country) + (1 | Age), data = data, REML = FALSE).

To facilitate interpretation of results, we subsequently split the data by sex of rater and re-ran the models. Table 3 displays fixed effects for the country-level model, for both sexes combined and for each sex separately. Full model results including random effects are given in Supplementary Table S2 in the Supplementary Materials; Models excluding participants on the basis of their reported sexual orientation and/or age are given in Supplementary Tables S3, S4, and show the same pattern of results.

**Table 3:** Model 1: Linear mixed effects model testing for differences in assortative preferences for mate height as a function of a short-term versus long-term relationship context, nesting participants by country.

	Women and Men	Women only	Men only
(Intercept)	−0.103	1.167^***^	−1.244^*^
	[−0.678, 0.472]	[0.612, 1.722]	[−2.318, −0.169]
Own height (of rater, z-scored)	1.892^***^	2.461^***^	1.453^**^
	[1.304, 2.479]	[1.700, 3.222]	[0.519, 2.388]
Sex (of rater)	−2.374^***^		
	[−3.524, −1.224]		
Relationship context	0.112	0.392	−0.140
	[−0.462, 0.686]	[−0.169, 0.953]	[−1.249, 0.969]
Own height × Sex	−0.681		
	[−1.856, 0.494]		
Own height × Relationship context	0.735^*^	0.381	1.076^*^
	[0.172, 1.299]	[−0.265, 1.027]	[0.114, 2.038]
Sex × Relationship context	−0.558		
	[−1.707, 0.590]		
Own height × Sex × Relationship context	0.690		
	[−0.437, 1.816]		

Own height of rater is coded in models as “height_z_scored”; Sex of rater is coded as “Sex”; Relationship context has two levels: short-term, long-term; [Lower 95% CI, Upper 95% CI].

See Supplementary Table S2 for full model with random effects.

*** *p < *0.001;

** *p < *0.01;

* *p < *0.05.

**Figure 2:**
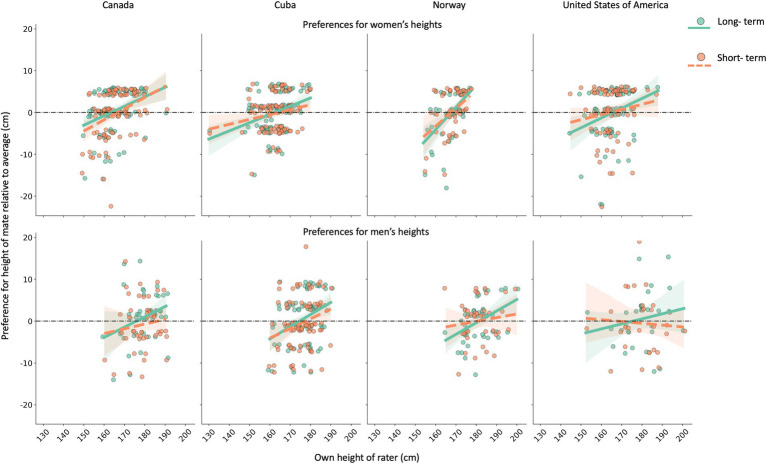
Assortative preferences for the heights of potential female mates (top row) and male mates (bottom row) in all four countries. The heights of raters positively predict how much taller-than-average or shorter-than-average they prefer their mates to be, with similar effects observed across countries. Men’s preferences for women’s heights are relatively stronger for a long-term (solid lines and green coloration) than short-term (dashed lines and orange coloration) relationship context. 95% confidence intervals are represented by shaded regions surrounding each regression line.

The results of Model 1 show that, in general, men preferred shorter women whereas women preferred taller men, relative to their own body heights (Table 3). Examining these effects for each sex separately while controlling for country-level variance shows that, on average, women prefer men 2.3 cm (or almost 1 inch) taller than the average men in their country, and men prefer women 2.5 cm (or about 1 inch) shorter than the average women in their country. Own height therefore positively predicted preferences for mate height, confirming positive assortative preferences for height in both sexes and across countries (Figure 2). However, this was qualified by relationship context in preferences for women’s heights. Indeed, taller men preferred relatively shorter women for short-term relationships than they did for long-term relationships (Table 3; Figure 2).

We then tested the same model again, replacing country with the self-reported free-response nationality (*k* = 39) of each participant, rather than their country of residence (see Supplementary Table S1 for a break-down of reported nationalities).

The R code for Model 2 nesting participants by nationality is:

lmer(height_preference ~ height_z_scored × Sex × Relationship_context + (1 + height_z_scored × Sex Relationship_context || ID_NUMBER,Nationality_selfreport,Country) + (1 | Age), data = data, REML = FALSE).

Table 4 displays fixed-effects for the nationality-level model, comparing men and women together and separately. Full model results including random effects are given in Supplementary Table S5 in the Supplementary Materials. Models excluding participants on the basis of their reported sexual orientation and/or age are given in Supplementary Tables S6, S7, and show the same pattern of results.

**Table 4:** Model 2: Linear mixed effects model testing for differences in assortative preferences for mate height as a function of a short-term versus long-term relationship context, nesting participants by self-reported nationality.

	Women and Men	Women only	Men only
(Intercept)	−0.020	1.041^***^	−1.015
	[−0.571, 0.530]	[0.498, 1.584]	[−2.046, 0.015]
Own height (of rater, z-scored)	1.649^***^	2.207^***^	1.189^*^
	[1.095, 2.202]	[1.502, 2.912]	[0.293, 2.086]
Sex (of rater)	−2.033^***^		
	[−3.133, −0.932]		
Relationship context	0.111	0.386	−0.138
	[−0.463, 0.685]	[−0.176, 0.947]	[−1.247, 0.970]
Own height × Sex	−0.815		
	[−1.921, 0.291]		
Own height × Relationship context	0.743^*^	0.390	1.076^*^
	[0.180, 1.305]	[−0.256, 1.035]	[0.115, 2.038]
Sex × Relationship context	−0.551		
	[−1.699, 0.597]		
Own height × Sex × Relationship context	0.675		
	[−0.451, 1.800]		

Own height of rater is coded as “height_z_scored”; Sex of rater is coded as “Sex”; Relationship context has two levels: short-term, long-term; [Lower 95% CI, Upper 95% CI].

See Supplementary Table S5 for full model with random effects.

*** *p < *0.001; and

* *p < *0.05.

In Model 2, own height again significantly and positively predicted preferences for height. As found in the country-level model, there was a main effect of relationship context, specific to preferences for women’s heights. This was qualified by an interaction with the rater’s own height, revealing again that for short-term relationships taller men preferred relatively shorter women than they did for long-term relationships (Table 4; Figure 2).

## Discussion

Our results partially corroborate previous findings, namely that women generally prefer taller men, and men generally prefer shorter women, relative to their own heights (Beigel, 1954; Salska et al., 2008; Courtiol et al., 2010; Stulp et al., 2013a). This result extends the “male taller norm” to four industrialized countries. Our results further corroborate a positive relationship between participants’ own heights and their mate height preferences, that is, positive assortative preferences for height (Stulp et al., 2017). However, assortative height preferences were qualified by relationship-context. Surprisingly, and in contrast to research on individual differences in preferences for facial and vocal masculinity in the context of a short-term versus long-term relationship (Little et al., 2011), our effects were specific to preferences for women’s heights. Taller men preferred relatively shorter women for short-term relationships than for long-term relationships.

Few studies have examined men’s preferences for women’s heights, and those studies have typically produced smaller effect sizes and less consistent results than have studies examining women’s preferences for men’s heights (Pierce, 1996; Salska et al., 2008; Courtiol et al., 2010; Stulp et al., 2013a). In addition, no previous study, to the authors knowledge, has tested for differences in men’s preferences for women’s heights across relationship contexts, despite the important trade-off between female height and reproduction (Stearns, 2000; Stulp and Barrett, 2016). However, our finding that women’s preferences for men’s heights did not differ (while men’s did) is unexpected, as predictions regarding context-specific mating strategies have traditionally focused on variation in women’s preferences (Buss and Schmitt, 1993, 2019). Moreover, one previous study found that women do prefer a larger sexual dimorphism in stature (SDS) for hypothetical short-term than long-term relationships (i.e., they prefer silhouette drawings representing a relatively larger difference in height between a male–female pair; Pawlowski and Jasienska, 2005). However, that study used the SDS scale (Pawlowski, 2003), aimed at measuring preferences for relative height between heterosexual pairs, that is not directly comparable to our scales. While our sample size of female raters was more than double that of Pawlowski and Jasienska (2005), suggesting that a lack of statistical power in our study is unlikely to explain the contrasting results, their study controlled for phase of menstrual cycle in their female raters whereas our study did not. Regrettably, neither study examined the potential influence of the current relationship status of participants on height preferences. More research is clearly needed to understand context-specific mating strategies in height preferences, particularly for women’s heights, using comparable methods to discern the robustness of these effects.

The absolute height preferences of each sex observed here support a general tendency for raters to prefer taller-than-average men and shorter-than-average women and, most consistently, to dis-prefer mates of either sex that exhibit a very short or very tall stature (Courtiol et al., 2010; Stulp and Barrett, 2016 for reviews). These observed absolute preferences corroborate studies suggesting that the costs of shortness outweigh the benefits of tallness, particularly for men (Stulp et al., 2014), wherein both men and women of average to somewhat above-average height appear to enjoy the highest reproductive success, at least in western societies, though this relationship varies widely across studies (Stulp and Barrett, 2016).

Despite modest cross-cultural differences in the population-level height distributions of Cubans, North Americans, and Norwegians, raters from all countries sampled in this study showed a similar pattern of height preferences. A recent meta-analysis comparing the heights of actual mated heterosexual couples also found no significant cultural differences (Stulp et al., 2017), suggesting that individual height differences may trump population-level height differences, as the variation in heights within countries is typically several times greater than between countries. Fink et al. (2007) also found no cross-cultural differences in preferences for sexual dimorphism in stature among participants from Austria, Germany and the United Kingdom, and no differences when comparing these samples to a Polish sample (Pawlowski, 2003).

While previous studies have shown relatively stronger preferences for various physical traits in hypothetical short-term compared to long-term relationship contexts, particularly for androgen-mediated masculinity in the face, body and voice (Little et al., 2011; Puts et al., 2012), our results suggest that this ostensibly adaptive mechanism may not robustly or consistently generalize to women’s preferences for all sexually dimorphic male traits, including physical height. However, while height is not closely linked to androgen levels in men (Kowal et al., 2021), preferences for indices of mate quality that are not directly hormonally mediated (such as height) may nevertheless differ for short-term versus long-term mates (Buss et al., 1990; Regan et al., 2000; Muggleton and Fincher, 2017). We did not find evidence of this for preferences of men’s heights.

Like the SDS scale (Pawlowski, 2003; Pawlowski and Jasienska, 2005), the scale used in the present study represented a normal distribution of heights for the sampled populations, omitting extremes, and importantly, did not elicit floor or ceiling effects. However, a limitation of such a scale is that it constrains the extent to which very short or very tall respondents can choose partners who are much shorter or taller than themselves. Thus, a broader representation of heights would allow for a correspondingly broader range of preference responses, and potentially a stronger mapping between own and preferred height. Incorporating a broader range of height preference options would also allow researchers to more readily use a single standardized height preference scale when comparing responses across human populations whose height distributions vary considerably from one another. Another potential limitation of the scale employed here is that visual representations of bodies were given alongside a numeric metric representing each figure’s height. While this allowed us to quantify height preferences in objective units, it prevents conclusions about whether participants were basing their judgments on the illustration, the metric, or both. The commonly used SDS scale does not contain metrics (Pawlowski and Jasienska, 2005; Fink et al., 2007; Sorokowski et al., 2011; Valentova et al., 2014), and that approach may indeed increase the implicit nature of the preference task and reduce conscious cognitive biases. Finally, while physical height and weight measurements were taken from our Canadian, Cuban and Norwegian participants (80% of our study sample), American respondents self-reported their body size. Self-reports of height can be biased, particularly among men (Merrill and Richardson, 2009), and thus have the potential to weaken the true association between own and preferred height, as may have been the case for our sample of American men.

Despite the cross-cultural nature of this study, the countries sampled here all score high on the World Health Organization’s Global Health Statistics, with comparably low rates of disease and childhood mortality (World Health Statistics, 2016). It is possible that cross-cultural differences in mate height preferences across relationship contexts may differ in countries with a high versus low health index. For example, selection may favor earlier sexual maturation (and thus shorter stature) in countries with high childhood mortality and low life expectancy (Sear, 2010). Indeed, assortative preferences for height have not been consistently replicated in non-traditional African societies namely among the Hadza, Himba, Datoga and Tsimane’ tribes (Sear and Marlowe, 2009; Sorokowski et al., 2011, 2015; Sorokowski and Butovskaya, 2012). Replication studies may therefore include participants from countries representing a wider range of ecological conditions. In future work, researchers may also test whether the preferences observed here translate into real-life mate choices, that is, whether or not stronger assortative mating in height is observed in actual committed (e.g., married) couples compared to shorter-term (casual) partnerships. Finally, it is important to emphasize that human relationships are complex and that mating contexts extend beyond a binary division of short-term and long-term partners. Integrating this complexity in study designs will undoubtedly help to illuminate and advance our understanding of human mate preferences.

## Data availability statement

The datasets analyzed for this study, full statistical models, R code and output, and research stimuli can be found in the online Supplementary materials and the Open Science Framework (https://osf.io/ah97w/).

## Ethics statement

The studies involving human participants were reviewed and approved by the McMaster Research Ethics Board and methods were carried out in accordance with the approved guidelines. The participants provided their written informed consent to participate in this study.

## Author contributions

KP and DF designed the research. KP, MF-A, ND-S, AO, AS, and RP collected the data. KP, AO, and RP coded and analyzed the data. DF performed statistical modeling. KP created stimuli and figures. KP, AO, and DF wrote the paper. KP, NE, EM, CL, and DF supervised the project. All authors contributed to the article and approved the submitted version.

## Funding

This cross-cultural research was funded by the Ministry of Science and Higher Education (Iuventus grant #0619/IP3/2016/74 to KP), the Social Sciences and Humanities Research Council of Canada (Michael Smith Foreign Study Supplement 771-2013-0108 to KP), a Centre National de la Recherche Scientifique 80-Prime grant (EvoHuman), and the University of Tennessee’s Center for Sensory Science (sensory.tennessee.edu, United States). AO was supported by a Scholarship for Outstanding Young Academics (#626/STYP/12/2017).

## Conflict of interest

The authors declare that the research was conducted in the absence of any commercial or financial relationships that could be construed as a potential conflict of interest.

## Publisher’s note

All claims expressed in this article are solely those of the authors and do not necessarily represent those of their affiliated organizations, or those of the publisher, the editors and the reviewers. Any product that may be evaluated in this article, or claim that may be made by its manufacturer, is not guaranteed or endorsed by the publisher.
