# Supplementary material for: Assortative mate preferences for height across short-term and long-term relationship contexts in a cross-cultural sample
Source: Front Psychol. 2022 Aug 25;13:937146. doi: 10.3389/fpsyg.2022.937146 (PMC9454610; doi:10.3389/fpsyg.2022.937146)
Supplement: Supplementary file 1 [file Data_Sheet_1.PDF]

Assortative Preferences for Height

Assortative preferences for height

==============================

What are we testing?

- Does own height predict height of image chosen?
- Do taller people prefer taller partners, and shorter people prefer shorter partners?
- Does preference vary depending on relationship context (short term/long term)
- Does the sex of the rater predict their preference for height?
- Do any relationships survive controlling for age

==============================

Load packages and data

```
library(lmerTest)
```

```
## Loading required package: lme4
```

```
## Loading required package: Matrix
```

```
##
## Attaching package: 'lmerTest'
```

```
## The following object is masked from 'package:lme4':
##
##     lmer
```

```
## The following object is masked from 'package:stats':
##
##     step
```

```
library(tidyverse)
```

```
## ── Attaching packages ─────────────────────────────────────── tidyverse 1.3.1 ──
```

```
## ✓ ggplot2 3.3.6     ✓ purrr   0.3.4
## ✓ tibble  3.1.6     ✓ dplyr   1.0.7
## ✓ tidyr   1.1.4     ✓ stringr 1.4.0
## ✓ readr   2.1.1     ✓ forcats 0.5.1
```

```
## ── Conflicts ────────────────────────────────────────── tidyverse_conflicts() ──
## x tidyr::expand() masks Matrix::expand()
## x dplyr::filter() masks stats::filter()
## x dplyr::lag()    masks stats::lag()
## x tidyr::pack()   masks Matrix::pack()
## x tidyr::unpack() masks Matrix::unpack()
```

```
library(psycho)
```

```
##
## Attaching package: 'psycho'
```

```
## The following object is masked from 'package:lme4':
##
##     golden
```

```
library(emmeans)
library(ggbeeswarm)
library(sjPlot)
library(jtools)

rawdata <- read_csv('Assort height pref - dataset LMM 06.2019.csv')
```

```
## Rows: 1072 Columns: 13
```

```
## ── Column specification ────────────────────────────────────────────────────────
## Delimiter: ","
## chr (7): ID, Sex, Country, Nationality_selfreport, Preferredsex_selfreport, ...
## dbl (6): ID_NUMBER, Age, Height, Weight, ethnicity_coded, HEIGHTpref_CM
```

```
##
## ℹ Use `spec()` to retrieve the full column specification for this data.
## ℹ Specify the column types or set `show_col_types = FALSE` to quiet this message.
```

```
head(rawdata)
```

```
## # A tibble: 6 × 13
##   ID_NUMBER ID     Sex     Age Height Weight Country Nationality_selfreport
##       <dbl> <chr>  <chr> <dbl>  <dbl>  <dbl> <chr>   <chr>
## 1         1 4385Ca Male     19   177    56.5 Canada  <NA>
## 2         1 4385Ca Male     19   177    56.5 Canada  <NA>
## 3         2 4388Ca Male     18   186.   81.5 Canada  <NA>
## 4         2 4388Ca Male     18   186.   81.5 Canada  <NA>
## 5         3 4443Ca Male     18   179    NA   Canada  <NA>
## 6         3 4443Ca Male     18   179    NA   Canada  <NA>
## # … with 5 more variables: ethnicity_coded <dbl>,
## #   Preferredsex_selfreport <chr>, Relationship_context <chr>,
## #   HEIGHTpref_ORG <chr>, HEIGHTpref_CM <dbl>
```

==

Z-score height

```
data <- rawdata[complete.cases(rawdata), ]
data <- data %>%
  mutate(
    height_z_scored = (Height - mean(Height))/sd(Height)
  ) %>%
  mutate(Sex = recode(Sex, "Female" = -0.5, "Male" = 0.5)) %>%
  mutate(Relationship_context = recode(Relationship_context, "ShortTerm" = -0.5, "LongTerm" = 0.5))
```

==

Centre height preference

```
data <- data %>%
    group_by(Country, Sex) %>%
    mutate(
      height_preference = HEIGHTpref_CM - mean(HEIGHTpref_CM)
      ) %>%
    ungroup()
```

The following analyses test what happens if we exclude people who did reported sexual preferences for the opposite sex and who are 40 years old or under.

This was suggested by Reviewer 1.

===============================

Model 1

Model 1 without exclusions

```
by_country_model_no_exclusions = lmer(height_preference ~ height_z_scored * Sex * Relationship_context  + (1 + he
ight_z_scored * Sex * Relationship_context || ID_NUMBER:Country) + (1 | Age), data=data, REML=FALSE)
```

```
## boundary (singular) fit: see ?isSingular
```

```
## Warning: Model failed to converge with 4 negative eigenvalues: -1.0e-04 -2.1e-04
## -4.5e-04 -4.0e-03
```

```
summ(by_country_model_no_exclusions, confint=TRUE, digits=3)
```

```
## Warning in summ.merMod(by_country_model_no_exclusions, confint = TRUE, digits = 3): Could not calculate r-squa
red. Try removing missing data
## before fitting the model.
```

**Observations**	1008
**Dependent variable**	height_preference
**Type**	Mixed effects linear regression

**AIC**	6021.895
**BIC**	6110.378

Fixed Effects

	Est.	2.5%	97.5%	t val.	d.f.	p
**(Intercept)**	-0.103	-0.678	0.472	-0.351	429.536	0.726
**height_z_scored**	1.892	1.304	2.479	6.312	240.179	0.000
**Sex**	-2.374	-3.524	-1.224	-4.046	429.536	0.000
**Relationship_context**	0.112	-0.462	0.686	0.383	459.910	0.702
**height_z_scored:Sex**	-0.681	-1.856	0.494	-1.136	240.179	0.257

p values calculated using Satterthwaite d.f.

Fixed Effects

	Est.	2.5%	97.5%	t val.	d.f.	p
**height_z_scored:Relationship_context**	0.735	0.172	1.299	2.558	227.389	0.011
**Sex:Relationship_context**	-0.558	-1.707	0.590	-0.953	459.910	0.341
**height_z_scored:Sex:Relationship_context**	0.690	-0.437	1.816	1.200	227.389	0.232

p values calculated using Satterthwaite d.f.

Random Effects

Group	Parameter	Std. Dev.
ID_NUMBER.Country	(Intercept)	4.252
ID_NUMBER.Country.1	height_z_scored	1.353
ID_NUMBER.Country.2	Sex	0.000
ID_NUMBER.Country.3	Relationship_context	3.303
ID_NUMBER.Country.4	height_z_scored:Sex	1.425
ID_NUMBER.Country.5	height_z_scored:Relationship_context	0.784
ID_NUMBER.Country.6	Sex:Relationship_context	1.497
ID_NUMBER.Country.7	height_z_scored:Sex:Relationship_context	1.130
Age	(Intercept)	0.000
Residual		2.158

Grouping Variables

Group	# groups	ICC
ID_NUMBER:Country	514	0.434
Age	50	0.044

Model 1 with both exclusions

```
by_country_model_exclusions = lmer(height_preference ~ height_z_scored * Sex * Relationship_context + (1 + height
_z_scored * Sex * Relationship_context || ID_NUMBER:Country) + (1 | Age), data=subset(data, Preferredsex_selfrepo
rt == 'PrefersOppositeSex' & Age <=40), REML=FALSE)
```

```
## boundary (singular) fit: see ?isSingular
```

```
## Warning: Model failed to converge with 3 negative eigenvalues: -7.0e-04 -1.8e-03
## -2.4e-03
```

```
summ(by_country_model_exclusions, confint=TRUE, digits=3)
```

```
## Warning in summ.merMod(by_country_model_exclusions, confint = TRUE, digits = 3): Could not calculate r-square
d. Try removing missing data
## before fitting the model.
```

**Observations**	828
**Dependent variable**	height_preference
**Type**	Mixed effects linear regression

**AIC**	4812.393
**BIC**	4897.335

Fixed Effects

	Est.	2.5%	97.5%	t val.	d.f.	p
**(Intercept)**	-0.123	-0.666	0.421	-0.443	341.767	0.658
**height_z_scored**	1.918	1.335	2.502	6.442	270.475	0.000
**Sex**	-3.486	-4.573	-2.398	-6.283	341.767	0.000
**Relationship_context**	0.171	-0.434	0.776	0.555	346.191	0.579
**height_z_scored:Sex**	-0.601	-1.768	0.566	-1.009	270.475	0.314
**height_z_scored:Relationship_context**	0.728	0.099	1.357	2.268	231.591	0.024
**Sex:Relationship_context**	-0.504	-1.714	0.706	-0.816	346.191	0.415
**height_z_scored:Sex:Relationship_context**	0.769	-0.488	2.027	1.199	231.591	0.232

p values calculated using Satterthwaite d.f.

Random Effects

Group	Parameter	Std. Dev.

Random Effects

Group	Parameter	Std. Dev.
ID_NUMBER.Country	(Intercept)	3.398
ID_NUMBER.Country.1	height_z_scored	1.531
ID_NUMBER.Country.2	Sex	0.126
ID_NUMBER.Country.3	Relationship_context	2.237
ID_NUMBER.Country.4	height_z_scored:Sex	1.520
ID_NUMBER.Country.5	height_z_scored:Relationship_context	1.360
ID_NUMBER.Country.6	Sex:Relationship_context	2.337
ID_NUMBER.Country.7	height_z_scored:Sex:Relationship_context	1.200
Age	(Intercept)	0.000
Residual		2.454

Grouping Variables

Group	# groups	ICC
ID_NUMBER:Country	422	0.321
Age	25	0.065

Model 1 with excluding people based on sexual orientation, but not age

```
by_country_model_sexual_orientation_exclusion = lmer(height_preference ~ height_z_scored * Sex * Relationship_context + (1 + height_z_scored * Sex * Relationship_context || ID_NUMBER:Country) + (1 | Age), data=subset(data, Preferredsex_selfreport == 'PrefersOppositeSex'), REML=FALSE)
```

```
## boundary (singular) fit: see ?isSingular
```

```
## Warning: Model failed to converge with 2 negative eigenvalues: -1.0e-05 -2.9e-05
```

```
summ(by_country_model_sexual_orientation_exclusion, confint=TRUE, digits=3)
```

```
## Warning in summ.merMod(by_country_model_sexual_orientation_exclusion, confint = TRUE, : Could not calculate r-squared. Try removing missing data
## before fitting the model.
```

**Observations**	940
**Dependent variable**	height_preference
**Type**	Mixed effects linear regression

**AIC**	5479.895
**BIC**	5567.120

Fixed Effects

	Est.	2.5%	97.5%	t val.	d.f.	p
**(Intercept)**	-0.128	-0.646	0.391	-0.482	387.855	0.630
**height_z_scored**	1.910	1.354	2.466	6.736	298.666	0.000
**Sex**	-3.248	-4.285	-2.210	-6.135	387.855	0.000
**Relationship_context**	0.184	-0.407	0.775	0.610	432.040	0.542
**height_z_scored:Sex**	-0.571	-1.683	0.540	-1.008	298.666	0.314
**height_z_scored:Relationship_context**	0.740	0.171	1.308	2.549	167.693	0.012
**Sex:Relationship_context**	-0.593	-1.775	0.589	-0.983	432.040	0.326
**height_z_scored:Sex:Relationship_context**	0.591	-0.546	1.729	1.019	167.693	0.310

p values calculated using Satterthwaite d.f.

Random Effects

Group	Parameter	Std. Dev.
ID_NUMBER.Country	(Intercept)	3.384
ID_NUMBER.Country.1	height_z_scored	1.335
ID_NUMBER.Country.2	Sex	0.002
ID_NUMBER.Country.3	Relationship_context	2.479
ID_NUMBER.Country.4	height_z_scored:Sex	2.527
ID_NUMBER.Country.5	height_z_scored:Relationship_context	0.412

Random Effects		
**Group**	**Parameter**	**Std. Dev.**
ID_NUMBER.Country.6	Sex:Relationship_context	2.939
ID_NUMBER.Country.7	height_z_scored:Sex:Relationship_context	1.142
Age	(Intercept)	0.000
Residual		2.466

Grouping Variables		
**Group**	**# groups**	**ICC**
ID_NUMBER:Country	479	0.273
Age	50	0.042

Model 1 with excluding people based on age, but no sexual orientation

```
by_country_model_age_exclusion = lmer(height_preference ~ height_z_scored * Sex * Relationship_context + (1 + hei
ght_z_scored * Sex * Relationship_context || ID_NUMBER:Country) + (1 | Age), data=subset(data, Age<=40), REML=FAL
SE)
```

```
## boundary (singular) fit: see ?isSingular
```

```
## Warning: Model failed to converge with 2 negative eigenvalues: -2.6e-04 -3.3e-04
```

```
summ(by_country_model_age_exclusion, confint=TRUE, digits=3)
```

```
## Warning in summ.merMod(by_country_model_age_exclusion, confint = TRUE, digits = 3): Could not calculate r-squa
red. Try removing missing data
## before fitting the model.
```

**Observations**	892
**Dependent variable**	height_preference
**Type**	Mixed effects linear regression

**AIC**	5303.676
**BIC**	5389.958

Fixed Effects

	Est.	2.5%	97.5%	t val.	d.f.	p
**(Intercept)**	-0.073	-0.669	0.522	-0.242	385.823	0.809
**height_z_scored**	1.840	1.234	2.447	5.947	220.265	0.000
**Sex**	-2.557	-3.748	-1.367	-4.210	385.823	0.000
**Relationship_context**	0.120	-0.465	0.706	0.403	375.119	0.687
**height_z_scored:Sex**	-0.596	-1.808	0.617	-0.962	220.265	0.337
**height_z_scored:Relationship_context**	0.692	0.078	1.307	2.209	265.582	0.028
**Sex:Relationship_context**	-0.475	-1.645	0.696	-0.794	375.119	0.427
**height_z_scored:Sex:Relationship_context**	0.827	-0.402	2.056	1.319	265.582	0.188

p values calculated using Satterthwaite d.f.

Random Effects

Group	Parameter	Std. Dev.
ID_NUMBER.Country	(Intercept)	2.897
ID_NUMBER.Country.1	height_z_scored	1.130
ID_NUMBER.Country.2	Sex	6.247
ID_NUMBER.Country.3	Relationship_context	2.807
ID_NUMBER.Country.4	height_z_scored:Sex	1.186
ID_NUMBER.Country.5	height_z_scored:Relationship_context	1.141
ID_NUMBER.Country.6	Sex:Relationship_context	3.244
ID_NUMBER.Country.7	height_z_scored:Sex:Relationship_context	2.262
Age	(Intercept)	0.000
Residual		2.032

Grouping Variables

Grouping Variables

Group	# groups	ICC
ID_NUMBER:Country	455	0.106
Age	25	0.016

```
export_summs(by_country_model_no_exclusions, by_country_model_exclusions, by_country_model_sexual_orientation_exc
lusion, by_country_model_age_exclusion, ci_level = 0.95, statistics = "all", digits=3, error_format = "[{conf.lo
w}, {conf.high}]", model.names = list("Full Data", "Excluding based on sexual orientation and age", "Excluding ba
sed only on sexual orientation", "Excluding based only on age"), to.file='Word', file.name='~/work/Papers/Assorta
tiveHeight/Frontierstable_model1_exclusion_comparison.docx')
```

```
## Warning in (function (model, scale = FALSE, confint = getOption("summ-confint", : Could not calculate r-square
d. Try removing missing data
## before fitting the model.
```

```
## Warning in (function (model, scale = FALSE, confint = getOption("summ-confint", : Could not calculate r-square
d. Try removing missing data
## before fitting the model.
```

```
## Warning in (function (model, scale = FALSE, confint = getOption("summ-confint", : Could not calculate r-square
d. Try removing missing data
## before fitting the model.
```

```
## Warning in (function (model, scale = FALSE, confint = getOption("summ-confint", : Could not calculate r-square
d. Try removing missing data
## before fitting the model.
```

```
## Warning in summ.merMod(model = new("lmerModLmerTest", vcov_varpar = structure(c(12.5391294311154, : Could not
calculate r-squared. Try removing missing data
## before fitting the model.
```

```
## Warning in summ.merMod(model = new("lmerModLmerTest", vcov_varpar = structure(c(0.827674348159571, : Could not
calculate r-squared. Try removing missing data
## before fitting the model.
```

```
## Warning in summ.merMod(model = new("lmerModLmerTest", vcov_varpar = structure(c(45.7691811985266, : Could not
calculate r-squared. Try removing missing data
## before fitting the model.
```

```
## Warning in summ.merMod(model = new("lmerModLmerTest", vcov_varpar = structure(c(3577.57381646565, : Could not
calculate r-squared. Try removing missing data
## before fitting the model.
```

	Full Data	Excluding based on sexual orientation and age	Excluding based only on sexual orientation	Excluding based only on age
(Intercept)	-0.103	-0.123	-0.128	-0.073
	[-0.678, 0.472]	[-0.666, 0.421]	[-0.646, 0.391]	[-0.669, 0.522]
height_z_scored	1.892 ***	1.918 ***	1.910 ***	1.840 ***
	[1.304, 2.479]	[1.335, 2.502]	[1.354, 2.466]	[1.234, 2.447]
Sex	-2.374 ***	-3.486 ***	-3.248 ***	-2.557 ***
	[-3.524, -1.224]	[-4.573, -2.398]	[-4.285, -2.210]	[-3.748, -1.367]
Relationship_context	0.112	0.171	0.184	0.120
	[-0.462, 0.686]	[-0.434, 0.776]	[-0.407, 0.775]	[-0.465, 0.706]
height_z_scored:Sex	-0.681	-0.601	-0.571	-0.596
	[-1.856, 0.494]	[-1.768, 0.566]	[-1.683, 0.540]	[-1.808, 0.617]
height_z_scored:Relationship_context	0.735 *	0.728 *	0.740 *	0.692 *
	[0.172, 1.299]	[0.099, 1.357]	[0.171, 1.308]	[0.078, 1.307]
Sex:Relationship_context	-0.558	-0.504	-0.593	-0.475

	[-1.707, 0.590]	[-1.714, 0.706]	[-1.775, 0.589]	[-1.645, 0.696]
height_z_scored:Sex:Relationship_context	0.690	0.769	0.591	0.827
	[-0.437, 1.816]	[-0.488, 2.027]	[-0.546, 1.729]	[-0.402, 2.056]
nobs	1008	828	940	892
sigma	2.158	2.454	2.466	2.032
logLik	-2992.948	-2388.196	-2721.947	-2633.838
AIC	6021.895	4812.393	5479.895	5303.676
BIC	6110.378	4897.335	5567.120	5389.958
deviance	5985.895	4776.393	5443.895	5267.676
df.residual	990.000	810.000	922.000	874.000
p.value				
r.squared				
r.squared.fixed				
group.nobs.ID_NUMBER:Country	514.000	422.000	479.000	455.000
group.nobs.Age	50.000	25.000	50.000	25.000

*** $p < 0.001$; ** $p < 0.01$; * $p < 0.05$.

This table shows only the main effect of sex is different based on exclusion criteria, although it doesn't directly compare them in a significance test. The difference in models is based on excluding people who were not heterosexual from the model. This created a positive effect in the model where people were removed from the data based on sexual orientation. There is no way for us to know if this is a false positive brought on by reducing our statistical power, or if this because of a difference in opinion between these groups. This category is not just people who identify as LGBTQ2A+, but also people who prefer not to answer the question. Thus we will not make any claims about sexual orientation in our paper.

Break Model 1 down by sex

Women Full Model

```
women_by_country_model = lmer(height_preference ~ height_z_scored * Relationship_context  + (1 + height_z_scored
 * Relationship_context || ID_NUMBER:Country) + (1 | Age), data=subset(data, Sex == -0.5), REML=FALSE)
```

```
## Warning in checkConv(attr(opt, "derivs"), opt$par, ctrl = control$checkConv, :
## unable to evaluate scaled gradient
```

```
## Warning in checkConv(attr(opt, "derivs"), opt$par, ctrl = control$checkConv, :
## Model failed to converge: degenerate Hessian with 1 negative eigenvalues
```

```
summ(women_by_country_model, confint=TRUE, digits=3)
```

```
## Warning in summ.merMod(women_by_country_model, confint = TRUE, digits = 3): Could not calculate r-squared. Try
removing missing data
## before fitting the model.
```

**Observations**	633
**Dependent variable**	height_preference
**Type**	Mixed effects linear regression

**AIC**	3677.068
**BIC**	3721.573

Fixed Effects

	Est.	2.5%	97.5%	t val.	d.f.	p
**(Intercept)**	1.167	0.612	1.722	4.122	28.688	0.000
**height_z_scored**	2.461	1.700	3.222	6.337	166.772	0.000
**Relationship_context**	0.392	-0.169	0.953	1.369	303.643	0.172
**height_z_scored:Relationship_context**	0.381	-0.265	1.027	1.155	100.711	0.251

p values calculated using Satterthwaite d.f.

Random Effects

Group	Parameter	Std. Dev.
ID_NUMBER.Country	(Intercept)	3.472

Random Effects

Group	Parameter	Std. Dev.
ID_NUMBER.Country.1	height_z_scored	2.840
ID_NUMBER.Country.2	Relationship_context	2.640
ID_NUMBER.Country.3	height_z_scored:Relationship_context	0.761
Age	(Intercept)	0.116
Residual		2.285

Grouping Variables

Group	# groups	ICC
ID_NUMBER:Country	323	0.366
Age	43	0.245

Women Sexual Orientation & Age Exclusions

```
women_by_country_model_both_exclusions = lmer(height_preference ~ height_z_scored *  Relationship_context  + (1 +
height_z_scored * Relationship_context || ID_NUMBER:Country) + (1 | Age), data=subset(data, Age<=40 & Sex == -0.5
& Preferredsex_selfreport == 'PrefersOppositeSex' ), REML=FALSE)
```

```
## Warning in checkConv(attr(opt, "derivs"), opt$par, ctrl = control$checkConv, : Model is nearly unidentifiable:
large eigenvalue ratio
##  - Rescale variables?
```

```
## Warning: Model failed to converge with 1 negative eigenvalue: -1.5e-04
```

```
summ(women_by_country_model_both_exclusions, confint=TRUE, digits=3)
```

```
## Warning in summ.merMod(women_by_country_model_both_exclusions, confint = TRUE, : Could not calculate r-square
d. Try removing missing data
## before fitting the model.
```

**Observations**	520
**Dependent variable**	height_preference
**Type**	Mixed effects linear regression

**AIC**	2884.344
**BIC**	2926.882

Fixed Effects

	Est.	2.5%	97.5%	t val.	d.f.	p
**(Intercept)**	1.629	1.053	2.205	5.541	23.630	0.000
**height_z_scored**	2.340	1.648	3.032	6.626	160.881	0.000
**Relationship_context**	0.417	-0.154	0.987	1.432	221.921	0.153
**height_z_scored:Relationship_context**	0.329	-0.387	1.045	0.900	139.462	0.369

p values calculated using Satterthwaite d.f.

Random Effects

Group	Parameter	Std. Dev.
ID_NUMBER.Country	(Intercept)	2.854
ID_NUMBER.Country.1	height_z_scored	2.048
ID_NUMBER.Country.2	Relationship_context	1.792
ID_NUMBER.Country.3	height_z_scored:Relationship_context	1.666
Age	(Intercept)	0.441
Residual		2.236

Grouping Variables

Group	# groups	ICC
ID_NUMBER:Country	265	0.346
Age	23	0.178

Women Sexual Orientation but not Age Exclusion

```
women_by_country_model_sexual_orientation_exclusion = lmer(height_preference ~ height_z_scored *  Relationship_co
ntext  + (1 + height_z_scored * Relationship_context || ID_NUMBER:Country) + (1 | Age), data=subset(data, Sex ==
 -0.5 & Preferredsex_selfreport == 'PrefersOppositeSex' ), REML=FALSE)
```

```
## Warning in checkConv(attr(opt, "derivs"), opt$par, ctrl = control$checkConv, :
## unable to evaluate scaled gradient
```

```
## Warning in checkConv(attr(opt, "derivs"), opt$par, ctrl = control$checkConv, :
## Model failed to converge: degenerate Hessian with 1 negative eigenvalues
```

```
summ(women_by_country_model_sexual_orientation_exclusion, confint=TRUE, digits=3)
```

```
## Warning in summ.merMod(women_by_country_model_sexual_orientation_exclusion, : Could not calculate r-squared. T
ry removing missing data
## before fitting the model.
```

**Observations**	593
**Dependent variable**	height_preference
**Type**	Mixed effects linear regression

**AIC**	3331.034
**BIC**	3374.886

Fixed Effects

	Est.	2.5%	97.5%	t val.	d.f.	p
**(Intercept)**	1.478	0.944	2.013	5.421	35.979	0.000
**height_z_scored**	2.304	1.634	2.974	6.744	181.767	0.000
**Relationship_context**	0.481	-0.084	1.046	1.670	278.021	0.096
**height_z_scored:Relationship_context**	0.438	-0.227	1.104	1.291	113.618	0.199

p values calculated using Satterthwaite d.f.

Random Effects

Group	Parameter	Std. Dev.
ID_NUMBER.Country	(Intercept)	2.856
ID_NUMBER.Country.1	height_z_scored	2.234
ID_NUMBER.Country.2	Relationship_context	2.207
ID_NUMBER.Country.3	height_z_scored:Relationship_context	1.048

Random Effects		
**Group**	**Parameter**	**Std. Dev.**
Age	(Intercept)	0.424
Residual		2.356

Grouping Variables		
**Group**	**# groups**	**ICC**
ID_NUMBER:Country	302	0.328
Age	42	0.201

Women Age but not Sexual Orientation Exclusion

```
women_by_country_model_age_exclusion = lmer(height_preference ~ height_z_scored *  Relationship_context  + (1 + h
eight_z_scored * Relationship_context || ID_NUMBER:Country) + (1 | Age), data=subset(data, Age<=40 & Sex == -0.5
), REML=FALSE)
```

```
## boundary (singular) fit: see ?isSingular
```

```
summ(women_by_country_model_age_exclusion, confint=TRUE, digits=3)
```

```
## Warning in summ.merMod(women_by_country_model_age_exclusion, confint = TRUE, : Could not calculate r-squared.
Try removing missing data
## before fitting the model.
```

**Observations**	556
**Dependent variable**	height_preference
**Type**	Mixed effects linear regression

**AIC**	3181.647
**BIC**	3224.855

Fixed Effects						
	**Est.**	**2.5%**	**97.5%**	**t val.**	**d.f.**	**p**

p values calculated using Satterthwaite d.f.

Fixed Effects

	Est.	2.5%	97.5%	t val.	d.f.	p
**(Intercept)**	1.284	0.708	1.859	4.372	212.922	0.000
**height_z_scored**	2.331	1.577	3.085	6.061	147.146	0.000
**Relationship_context**	0.353	-0.214	0.920	1.219	241.075	0.224
**height_z_scored:Relationship_context**	0.262	-0.436	0.960	0.735	142.693	0.464

p values calculated using Satterthwaite d.f.

Random Effects

Group	Parameter	Std. Dev.
ID_NUMBER.Country	(Intercept)	3.469
ID_NUMBER.Country.1	height_z_scored	2.283
ID_NUMBER.Country.2	Relationship_context	2.075
ID_NUMBER.Country.3	height_z_scored:Relationship_context	1.568
Age	(Intercept)	0.000
Residual		2.255

Grouping Variables

Group	# groups	ICC
ID_NUMBER:Country	284	0.413
Age	24	0.179

```
export_summs(women_by_country_model, women_by_country_model_both_exclusions, women_by_country_model_sexual_orient
ation_exclusion, women_by_country_model_age_exclusion, ci_level = 0.95, statistics = "all", digits=3,  error_form
at = "[{conf.low}, {conf.high}]", model.names = list("Full Data", "Excluding based on sexual orientation and age"
, "Excluding based only on sexual orientation", "Excluding based only on age"), to.file='Word', file.name='~/wor
k/Papers/AssortativeHeight/Frontierstable_model1_exclusion_comparison_women_raters.docx')
```

```
## Warning in (function (model, scale = FALSE, confint = getOption("summ-confint", : Could not calculate r-square
d. Try removing missing data
## before fitting the model.
```

```
## Warning in (function (model, scale = FALSE, confint = getOption("summ-confint", : Could not calculate r-square
d. Try removing missing data
## before fitting the model.
```

```
## Warning in (function (model, scale = FALSE, confint = getOption("summ-confint", : Could not calculate r-square
d. Try removing missing data
## before fitting the model.
```

```
## Warning in (function (model, scale = FALSE, confint = getOption("summ-confint", : Could not calculate r-square
d. Try removing missing data
## before fitting the model.
```

```
## Warning in summ.merMod(model = new("lmerModLmerTest", vcov_varpar = structure(c(19982.1498417106, : Could not
calculate r-squared. Try removing missing data
## before fitting the model.
```

```
## Warning in summ.merMod(model = new("lmerModLmerTest", vcov_varpar = structure(c(0.0130398473697368, : Could no
t calculate r-squared. Try removing missing data
## before fitting the model.
```

```
## Warning in summ.merMod(model = new("lmerModLmerTest", vcov_varpar = structure(c(174814.103445142, : Could not
calculate r-squared. Try removing missing data
## before fitting the model.
```

```
## Warning in summ.merMod(model = new("lmerModLmerTest", vcov_varpar = structure(c(12267.1075780981, : Could not
calculate r-squared. Try removing missing data
## before fitting the model.
```

	Full Data	Excluding based on sexual orientation and age	Excluding based only on sexual orientation	Excluding based only on age
(Intercept)	1.167 ***	1.629 ***	1.478 ***	1.284 ***
	[0.612, 1.722]	[1.053, 2.205]	[0.944, 2.013]	[0.708, 1.859]
height_z_scored	2.461 ***	2.340 ***	2.304 ***	2.331 ***
	[1.700, 3.222]	[1.648, 3.032]	[1.634, 2.974]	[1.577, 3.085]

Relationship_context	0.392	0.417	0.481	0.353
	[-0.169, 0.953]	[-0.154, 0.987]	[-0.084, 1.046]	[-0.214, 0.920]
height_z_scored:Relationship_context	0.381	0.329	0.438	0.262
	[-0.265, 1.027]	[-0.387, 1.045]	[-0.227, 1.104]	[-0.436, 0.960]
nobs	633	520	593	556
sigma	2.285	2.236	2.356	2.255
logLik	-1828.534	-1432.172	-1655.517	-1580.823
AIC	3677.068	2884.344	3331.034	3181.647
BIC	3721.573	2926.882	3374.886	3224.855
deviance	3657.068	2864.344	3311.034	3161.647
df.residual	623.000	510.000	583.000	546.000
p.value				
r.squared				
r.squared.fixed				
group.nobs.ID_NUMBER:Country	323.000	265.000	302.000	284.000
group.nobs.Age	43.000	23.000	42.000	24.000

*** p < 0.001; ** p < 0.01; * p < 0.05.

Men Full Model, no Exclusions

```
men_by_country_model = lmer(height_preference ~ height_z_scored *  Relationship_context  + (1 + height_z_scored *
Relationship_context || ID_NUMBER:Country) + (1 | Age), data=subset(data, Sex == 0.5), REML=FALSE)
```

```
## boundary (singular) fit: see ?isSingular
```

```
## Warning: Model failed to converge with 1 negative eigenvalue: -1.5e+00
```

```
summ(men_by_country_model, confint=TRUE, digits=3)
```

```
## Warning in summm.merMod(men_by_country_model, confint = TRUE, digits = 3): Could not calculate r-squared. Try r
emoving missing data
## before fitting the model.
```

**Observations**	375
**Dependent variable**	height_preference
**Type**	Mixed effects linear regression

**AIC**	2325.887
**BIC**	2365.157

Fixed Effects

	Est.	2.5%	97.5%	t val.	d.f.	p
**(Intercept)**	-1.244	-2.318	-0.169	-2.268	189.945	0.024
**height_z_scored**	1.453	0.519	2.388	3.047	189.352	0.003
**Relationship_context**	-0.140	-1.249	0.969	-0.247	185.845	0.805
**height_z_scored:Relationship_context**	1.076	0.114	2.038	2.192	185.262	0.030

p values calculated using Satterthwaite d.f.

Random Effects

Group	Parameter	Std. Dev.
ID_NUMBER.Country	(Intercept)	4.661
ID_NUMBER.Country.1	height_z_scored	0.000
ID_NUMBER.Country.2	Relationship_context	2.925
ID_NUMBER.Country.3	height_z_scored:Relationship_context	0.000
Age	(Intercept)	0.000
Residual		3.121

Grouping Variables

Grouping Variables

Group	# groups	ICC
ID_NUMBER:Country	191	0.543
Age	35	0.000

Men Sexual Orientation & Age Exclusions

```
men_by_country_model_both_exclusions = lmer(height_preference ~ height_z_scored *  Relationship_context  + (1 + h
eight_z_scored * Relationship_context || ID_NUMBER:Country) + (1 | Age), data=subset(data, Age<=40 & Sex == 0.5 &
Preferredsex_selfreport == 'PrefersOppositeSex' ), REML=FALSE)
```

```
## boundary (singular) fit: see ?isSingular
```

```
summ(men_by_country_model_both_exclusions, confint=TRUE, digits=3)
```

```
## Warning in summ.merMod(men_by_country_model_both_exclusions, confint = TRUE, : Could not calculate r-squared.
Try removing missing data
## before fitting the model.
```

**Observations**	308
**Dependent variable**	height_preference
**Type**	Mixed effects linear regression

**AIC**	1895.662
**BIC**	1932.963

Fixed Effects

	Est.	2.5%	97.5%	t val.	d.f.	p
**(Intercept)**	-1.748	-2.834	-0.662	-3.154	155.311	0.002
**height_z_scored**	1.421	0.449	2.392	2.866	155.063	0.005
**Relationship_context**	-0.033	-1.264	1.197	-0.053	152.199	0.958
**height_z_scored:Relationship_context**	1.121	0.021	2.220	1.998	151.952	0.047

p values calculated using Satterthwaite d.f.

Random Effects

Group	Parameter	Std. Dev.
ID_NUMBER.Country	(Intercept)	4.293
ID_NUMBER.Country.1	height_z_scored	0.000
ID_NUMBER.Country.2	Relationship_context	3.392
ID_NUMBER.Country.3	height_z_scored:Relationship_context	0.000
Age	(Intercept)	0.000
Residual		2.965

Grouping Variables

Group	# groups	ICC
ID_NUMBER:Country	157	0.476
Age	23	0.000

Men Sexual Orientation but not Age Exclusion

```
men_by_country_model_sexual_orientation_exclusion = lmer(height_preference ~ height_z_scored *  Relationship_cont
ext  + (1 + height_z_scored * Relationship_context || ID_NUMBER:Country) + (1 | Age), data=subset(data, Sex == 0.
5 & Preferredsex_selfreport == 'PrefersOppositeSex'), REML=FALSE)
```

```
## boundary (singular) fit: see ?isSingular
```

```
## Warning: Model failed to converge with 1 negative eigenvalue: -2.2e-05
```

```
summ(men_by_country_model_sexual_orientation_exclusion, confint=TRUE, digits=3)
```

```
## Warning in summ.merMod(men_by_country_model_sexual_orientation_exclusion, : Could not calculate r-squared. Try
removing missing data
## before fitting the model.
```

**Observations**	347
**Dependent variable**	height_preference
**Type**	Mixed effects linear regression

**AIC**	2124.624
**BIC**	2163.117

Fixed Effects

	Est.	2.5%	97.5%	t val.	d.f.	p
**(Intercept)**	-1.664	-2.695	-0.633	-3.164	148.850	0.002
**height_z_scored**	1.475	0.572	2.377	3.203	101.062	0.002
**Relationship_context**	-0.089	-1.246	1.067	-0.151	171.926	0.880
**height_z_scored:Relationship_context**	1.016	0.018	2.014	1.996	171.259	0.048

p values calculated using Satterthwaite d.f.

Random Effects

Group	Parameter	Std. Dev.
ID_NUMBER.Country	(Intercept)	4.125
ID_NUMBER.Country.1	height_z_scored	0.543
ID_NUMBER.Country.2	Relationship_context	2.689
ID_NUMBER.Country.3	height_z_scored:Relationship_context	0.000
Age	(Intercept)	0.000
Residual		3.219

Grouping Variables

Group	# groups	ICC
ID_NUMBER:Country	177	0.487
Age	35	0.008

Men Age but not Sexual Orientation Exclusion

```
men_by_country_model_age_exclusion = lmer(height_preference ~ height_z_scored *  Relationship_context  + (1 + hei
ght_z_scored * Relationship_context || ID_NUMBER:Country) + (1 | Age), data=subset(data, Age<=40 & Sex == 0.5), R
EML=FALSE)
```

```
## boundary (singular) fit: see ?isSingular
```

```
summ(men_by_country_model_age_exclusion, confint=TRUE, digits=3)
```

```
## Warning in summm.merMod(men_by_country_model_age_exclusion, confint = TRUE, : Could not calculate r-squared. Tr
y removing missing data
## before fitting the model.
```

**Observations**	336
**Dependent variable**	height_preference
**Type**	Mixed effects linear regression

**AIC**	2096.737
**BIC**	2134.908

Fixed Effects

	Est.	2.5%	97.5%	t val.	d.f.	p
**(Intercept)**	-1.303	-2.437	-0.168	-2.250	169.673	0.026
**height_z_scored**	1.443	0.425	2.461	2.778	169.471	0.006
**Relationship_context**	-0.098	-1.264	1.067	-0.166	140.789	0.869
**height_z_scored:Relationship_context**	1.166	0.103	2.229	2.150	116.165	0.034

p values calculated using Satterthwaite d.f.

Random Effects

Group	Parameter	Std. Dev.
ID_NUMBER.Country	(Intercept)	4.587
ID_NUMBER.Country.1	height_z_scored	0.000
ID_NUMBER.Country.2	Relationship_context	1.715
ID_NUMBER.Country.3	height_z_scored:Relationship_context	0.808
Age	(Intercept)	0.000
Residual		3.558

Grouping Variables

Group	# groups	ICC
ID_NUMBER:Country	171	0.564
Age	23	0.000

```
export_summs(men_by_country_model, men_by_country_model_both_exclusions, men_by_country_model_sexual_orientation_
exclusion, men_by_country_model_age_exclusion, ci_level = 0.95, digits=3, statistics = "all", error_format = "[{c
onf.low}, {conf.high}]", model.names = list("Full Data", "Excluding based on sexual orientation and age", "Exclud
ing based only on sexual orientation", "Excluding based only on age"), to.file='Word', file.name='~/work/Papers/A
ssortativeHeight/Frontierstable_model1_exclusion_comparison_men_raters.docx')
```

```
## Warning in (function (model, scale = FALSE, confint = getOption("summ-confint", : Could not calculate r-square
d. Try removing missing data
## before fitting the model.
```

```
## Warning in (function (model, scale = FALSE, confint = getOption("summ-confint", : Could not calculate r-square
d. Try removing missing data
## before fitting the model.
```

```
## Warning in (function (model, scale = FALSE, confint = getOption("summ-confint", : Could not calculate r-square
d. Try removing missing data
## before fitting the model.
```

```
## Warning in (function (model, scale = FALSE, confint = getOption("summ-confint", : Could not calculate r-square
d. Try removing missing data
## before fitting the model.
```

```
## Warning in summ.merMod(model = new("lmerModLmerTest", vcov_varpar = structure(c(30395.3946842462, : Could not
calculate r-squared. Try removing missing data
## before fitting the model.
```

```
## Warning in summ.merMod(model = new("lmerModLmerTest", vcov_varpar = structure(c(3464.92256015057, : Could not
calculate r-squared. Try removing missing data
## before fitting the model.
```

```
## Warning in summ.merMod(model = new("lmerModLmerTest", vcov_varpar = structure(c(0.0146654435414699, : Could no
t calculate r-squared. Try removing missing data
## before fitting the model.
```

```
## Warning in summ.merMod(model = new("lmerModLmerTest", vcov_varpar = structure(c(273.236341542093, : Could not calculate r-squared. Try removing missing data
## before fitting the model.
```

	Full Data	Excluding based on sexual orientation and age	Excluding based only on sexual orientation	Excluding based only on age
(Intercept)	-1.244 *	-1.748 **	-1.664 **	-1.303 *
	[-2.318, -0.169]	[-2.834, -0.662]	[-2.695, -0.633]	[-2.437, -0.168]
height_z_scored	1.453 **	1.421 **	1.475 **	1.443 **
	[0.519, 2.388]	[0.449, 2.392]	[0.572, 2.377]	[0.425, 2.461]
Relationship_context	-0.140	-0.033	-0.089	-0.098
	[-1.249, 0.969]	[-1.264, 1.197]	[-1.246, 1.067]	[-1.264, 1.067]
height_z_scored:Relationship_context	1.076 *	1.121 *	1.016 *	1.166 *
	[0.114, 2.038]	[0.021, 2.220]	[0.018, 2.014]	[0.103, 2.229]
nobs	375	308	347	336
sigma	3.121	2.965	3.219	3.558
logLik	-1152.944	-937.831	-1052.312	-1038.368
AIC	2325.887	1895.662	2124.624	2096.737
BIC	2365.157	1932.963	2163.117	2134.908
deviance	2305.887	1875.662	2104.624	2076.737
df.residual	365.000	298.000	337.000	326.000
p.value				
r.squared				

r.squared.fixed				
group.nobs.ID_NUMBER:Country	191.000	157.000	177.000	171.000
group.nobs.Age	35.000	23.000	35.000	23.000

*** p < 0.001; ** p < 0.01; * p < 0.05.

```
export_summs(by_country_model_no_exclusions, women_by_country_model, men_by_country_model, ci_level = 0.95, stati
stics = "all", digits=3, error_format = "[{conf.low}, {conf.high}]", to.file='Word', model.names = list("Women an
d Men", "Women", "Men"), file.name='~/work/Papers/AssortativeHeight/Frontierstable_model1_compare_sexes.docx')
```

```
## Warning in (function (model, scale = FALSE, confint = getOption("summ-confint", : Could not calculate r-square
d. Try removing missing data
## before fitting the model.
```

```
## Warning in (function (model, scale = FALSE, confint = getOption("summ-confint", : Could not calculate r-square
d. Try removing missing data
## before fitting the model.
```

```
## Warning in (function (model, scale = FALSE, confint = getOption("summ-confint", : Could not calculate r-square
d. Try removing missing data
## before fitting the model.
```

```
## Warning in summ.merMod(model = new("lmerModLmerTest", vcov_varpar = structure(c(12.5391294311154, : Could not
calculate r-squared. Try removing missing data
## before fitting the model.
```

```
## Warning in summ.merMod(model = new("lmerModLmerTest", vcov_varpar = structure(c(19982.1498417106, : Could not
calculate r-squared. Try removing missing data
## before fitting the model.
```

```
## Warning in summ.merMod(model = new("lmerModLmerTest", vcov_varpar = structure(c(30395.3946842462, : Could not
calculate r-squared. Try removing missing data
## before fitting the model.
```

	Women and Men	Women	Men
(Intercept)	-0.103	1.167 ***	-1.244 *

	[-0.678, 0.472]	[0.612, 1.722]	[-2.318, -0.169]
height_z_scored	1.892 ***	2.461 ***	1.453 **
	[1.304, 2.479]	[1.700, 3.222]	[0.519, 2.388]
Sex	-2.374 ***		
	[-3.524, -1.224]		
Relationship_context	0.112	0.392	-0.140
	[-0.462, 0.686]	[-0.169, 0.953]	[-1.249, 0.969]
height_z_scored:Sex	-0.681		
	[-1.856, 0.494]		
height_z_scored:Relationship_context	0.735 *	0.381	1.076 *
	[0.172, 1.299]	[-0.265, 1.027]	[0.114, 2.038]
Sex:Relationship_context	-0.558		
	[-1.707, 0.590]		
height_z_scored:Sex:Relationship_context	0.690		
	[-0.437, 1.816]		
nobs	1008	633	375
sigma	2.158	2.285	3.121
logLik	-2992.948	-1828.534	-1152.944
AIC	6021.895	3677.068	2325.887
BIC	6110.378	3721.573	2365.157
deviance	5985.895	3657.068	2305.887
df.residual	990.000	623.000	365.000
p.value			
r.squared			

r.squared.fixed			
group.nobs.ID_NUMBER:Country	514.000	323.000	191.000
group.nobs.Age	50.000	43.000	35.000

*** p < 0.001; ** p < 0.01; * p < 0.05.

Estimated Marginal Means for Model

No exclusions Estimated marginal means and confidence intervals for each contrast level

nationality_model_sexual_orientation_exclusion, nationality_model_age_exclusion

```
emmeans(by_country_model_no_exclusions, "Sex", by="Relationship_context")
```

```
## boundary (singular) fit: see ?isSingular
```

```
## Warning: Model failed to converge with 2 negative eigenvalues: -6.7e-04 -6.5e-02
```

```
## NOTE: Results may be misleading due to involvement in interactions
```

```
## Relationship_context = -0.5:
##   Sex emmean    SE    df lower.CL upper.CL
##  -0.5  0.888 0.372  80.0    0.148   1.6284
##   0.5 -1.206 0.566 349.0   -2.319  -0.0936
##
## Relationship_context =  0.5:
##   Sex emmean    SE    df lower.CL upper.CL
##  -0.5  1.280 0.368  76.5    0.546   2.0131
##   0.5 -1.373 0.563 348.1   -2.481  -0.2660
##
## Degrees-of-freedom method: kenward-roger
## Confidence level used: 0.95
```

```
emmeans(by_country_model_no_exclusions, "Relationship_context", by="Sex")
```

```
## boundary (singular) fit: see ?isSingular
```

```
## Warning: Model failed to converge with 2 negative eigenvalues: -6.7e-04 -6.5e-02
```

```
## NOTE: Results may be misleading due to involvement in interactions
```

```
## Sex = -0.5:
##  Relationship_context emmean    SE    df lower.CL upper.CL
##                  -0.5  0.888 0.372  80.0    0.148   1.6284
##                   0.5  1.280 0.368  76.5    0.546   2.0131
##
## Sex =  0.5:
##  Relationship_context emmean    SE    df lower.CL upper.CL
##                  -0.5 -1.206 0.566 349.0   -2.319  -0.0936
##                   0.5 -1.373 0.563 348.1   -2.481  -0.2660
##
## Degrees-of-freedom method: kenward-roger
## Confidence level used: 0.95
```

```
emmeans(by_country_model_no_exclusions, "height_z_scored", by="Sex")
```

```
## boundary (singular) fit: see ?isSingular
```

```
## Warning: Model failed to converge with 2 negative eigenvalues: -6.7e-04 -6.5e-02
```

```
## NOTE: Results may be misleading due to involvement in interactions
```

```
## Sex = -0.5:
##  height_z_scored emmean    SE    df lower.CL upper.CL
##         1.38e-15   1.08 0.335  50.1    0.412    1.756
##
## Sex =  0.5:
##  height_z_scored emmean    SE    df lower.CL upper.CL
##         1.38e-15  -1.29 0.506 230.7   -2.288   -0.292
##
## Results are averaged over the levels of: Relationship_context
## Degrees-of-freedom method: kenward-roger
## Confidence level used: 0.95
```

```
emmeans(by_country_model_no_exclusions, "height_z_scored", by="Relationship_context")
```

```
## boundary (singular) fit: see ?isSingular
```

```
## Warning: Model failed to converge with 2 negative eigenvalues: -6.7e-04 -6.5e-02
```

```
## NOTE: Results may be misleading due to involvement in interactions
```

```
## Relationship_context = -0.5:
##  height_z_scored  emmean    SE   df lower.CL upper.CL
##         1.38e-15 -0.1590 0.344 75.3   -0.844    0.526
##
## Relationship_context =  0.5:
##  height_z_scored  emmean    SE   df lower.CL upper.CL
##         1.38e-15 -0.0469 0.342 74.3   -0.728    0.634
##
## Results are averaged over the levels of: Sex
## Degrees-of-freedom method: kenward-roger
## Confidence level used: 0.95
```

Model 1 - Both exclusions Estimated marginal means and confidence intervals for each contrast level

```
emmeans(by_country_model_exclusions, "Sex", by="Relationship_context")
```

```
## boundary (singular) fit: see ?isSingular
```

```
## Warning: Model failed to converge with 1 negative eigenvalue: -6.3e-05
```

```
## NOTE: Results may be misleading due to involvement in interactions
```

```
## Relationship_context = -0.5:
##   Sex emmean    SE    df lower.CL upper.CL
##  -0.5   1.34 0.361  58.9    0.618    2.063
##   0.5  -1.86 0.553 334.3   -2.948   -0.773
##
## Relationship_context =  0.5:
##   Sex emmean    SE    df lower.CL upper.CL
##  -0.5   1.75 0.357  55.4    1.037    2.467
##   0.5  -1.98 0.551 332.3   -3.062   -0.894
##
## Degrees-of-freedom method: kenward-roger
## Confidence level used: 0.95
```

```
emmeans(by_country_model_exclusions, "Relationship_context", by="Sex")
```

```
## boundary (singular) fit: see ?isSingular
```

```
## Warning: Model failed to converge with 1 negative eigenvalue: -6.3e-05
```

```
## NOTE: Results may be misleading due to involvement in interactions
```

```
## Sex = -0.5:
##  Relationship_context emmean    SE    df lower.CL upper.CL
##                  -0.5   1.34 0.361  58.9    0.618    2.063
##                   0.5   1.75 0.357  55.4    1.037    2.467
##
## Sex =  0.5:
##  Relationship_context emmean    SE    df lower.CL upper.CL
##                  -0.5  -1.86 0.553 334.3   -2.948   -0.773
##                   0.5  -1.98 0.551 332.3   -3.062   -0.894
##
## Degrees-of-freedom method: kenward-roger
## Confidence level used: 0.95
```

```
emmeans(by_country_model_exclusions, "height_z_scored", by="Sex")
```

```
## boundary (singular) fit: see ?isSingular
```

```
## Warning: Model failed to converge with 1 negative eigenvalue: -6.3e-05
```

```
## NOTE: Results may be misleading due to involvement in interactions
```

```
## Sex = -0.5:
##  height_z_scored emmean    SE    df lower.CL upper.CL
##          -0.0332   1.55 0.318  33.4     0.90    2.193
##
## Sex =  0.5:
##  height_z_scored emmean    SE    df lower.CL upper.CL
##          -0.0332  -1.92 0.484 205.2    -2.87   -0.966
##
## Results are averaged over the levels of: Relationship_context
## Degrees-of-freedom method: kenward-roger
## Confidence level used: 0.95
```

```
emmeans(by_country_model_exclusions, "height_z_scored", by="Relationship_context")
```

```
## boundary (singular) fit: see ?isSingular
```

```
## Warning: Model failed to converge with 1 negative eigenvalue: -6.3e-05
```

```
## NOTE: Results may be misleading due to involvement in interactions
```

```
## Relationship_context = -0.5:
##  height_z_scored emmean    SE   df lower.CL upper.CL
##          -0.0332 -0.260 0.333 69.8   -0.925    0.405
##
## Relationship_context =  0.5:
##  height_z_scored emmean    SE   df lower.CL upper.CL
##          -0.0332 -0.113 0.331 68.5   -0.774    0.548
##
## Results are averaged over the levels of: Sex
## Degrees-of-freedom method: kenward-roger
## Confidence level used: 0.95
```

Model 1 - Sexual Orientation exclusions Estimated marginal means and confidence intervals for each contrast level

```
emmeans(by_country_model_sexual_orientation_exclusion, "Sex", by="Relationship_context")
```

```
## boundary (singular) fit: see ?isSingular
```

```
## Warning: Model failed to converge with 3 negative eigenvalues: -2.9e-03 -4.6e-03
## -1.5e-02
```

```
## NOTE: Results may be misleading due to involvement in interactions
```

```
## Relationship_context = -0.5:
##   Sex emmean    SE    df lower.CL upper.CL
##  -0.5   1.24 0.342  82.6    0.558    1.918
##   0.5  -1.71 0.529 378.4   -2.745   -0.666
##
## Relationship_context =  0.5:
##   Sex emmean    SE    df lower.CL upper.CL
##  -0.5   1.71 0.338  77.8    1.042    2.386
##   0.5  -1.83 0.526 376.7   -2.861   -0.794
##
## Degrees-of-freedom method: kenward-roger
## Confidence level used: 0.95
```

```
emmeans(by_country_model_sexual_orientation_exclusion, "Relationship_context", by="Sex")
```

```
## boundary (singular) fit: see ?isSingular
```

```
## Warning: Model failed to converge with 3 negative eigenvalues: -2.9e-03 -4.6e-03
## -1.5e-02
```

```
## NOTE: Results may be misleading due to involvement in interactions
```

```
## Sex = -0.5:
##  Relationship_context emmean    SE    df lower.CL upper.CL
##                  -0.5   1.24 0.342  82.6    0.558    1.918
##                   0.5   1.71 0.338  77.8    1.042    2.386
##
## Sex =  0.5:
##  Relationship_context emmean    SE    df lower.CL upper.CL
##                  -0.5  -1.71 0.529 378.4   -2.745   -0.666
##                   0.5  -1.83 0.526 376.7   -2.861   -0.794
##
## Degrees-of-freedom method: kenward-roger
## Confidence level used: 0.95
```

```
emmeans(by_country_model_sexual_orientation_exclusion, "height_z_scored", by="Sex")
```

```
## boundary (singular) fit: see ?isSingular
```

```
## Warning: Model failed to converge with 3 negative eigenvalues: -2.9e-03 -4.6e-03
## -1.5e-02
```

```
## NOTE: Results may be misleading due to involvement in interactions
```

```
## Sex = -0.5:
##  height_z_scored emmean    SE    df lower.CL upper.CL
##         -0.00914   1.48 0.299  45.8    0.874    2.078
##
## Sex =  0.5:
##  height_z_scored emmean    SE    df lower.CL upper.CL
##         -0.00914  -1.77 0.460 227.6   -2.672   -0.861
##
## Results are averaged over the levels of: Relationship_context
## Degrees-of-freedom method: kenward-roger
## Confidence level used: 0.95
```

```
emmeans(by_country_model_sexual_orientation_exclusion, "height_z_scored", by="Relationship_context")
```

```
## boundary (singular) fit: see ?isSingular
```

```
## Warning: Model failed to converge with 3 negative eigenvalues: -2.9e-03 -4.6e-03
## -1.5e-02
```

```
## NOTE: Results may be misleading due to involvement in interactions
```

```
## Relationship_context = -0.5:
##  height_z_scored  emmean    SE   df lower.CL upper.CL
##         -0.00914 -0.2337 0.319 88.4   -0.867    0.399
##
## Relationship_context =  0.5:
##  height_z_scored  emmean    SE   df lower.CL upper.CL
##         -0.00914 -0.0565 0.316 87.1   -0.685    0.572
##
## Results are averaged over the levels of: Sex
## Degrees-of-freedom method: kenward-roger
## Confidence level used: 0.95
```

Model 1 - Age exclusions Estimated marginal means and confidence intervals for each contrast level

```
emmeans(by_country_model_age_exclusion, "Sex", by="Relationship_context")
```

```
## boundary (singular) fit: see ?isSingular
```

```
## Warning: Model failed to converge with 2 negative eigenvalues: -8.9e-06 -3.1e-05
```

```
## NOTE: Results may be misleading due to involvement in interactions
```

```
## Relationship_context = -0.5:
##   Sex emmean    SE    df lower.CL upper.CL
##  -0.5  0.991 0.391  58.7    0.209    1.773
##   0.5 -1.311 0.584 312.9   -2.460   -0.163
##
## Relationship_context =  0.5:
##   Sex emmean    SE    df lower.CL upper.CL
##  -0.5  1.343 0.387  56.0    0.568    2.119
##   0.5 -1.448 0.582 311.9   -2.593   -0.303
##
## Degrees-of-freedom method: kenward-roger
## Confidence level used: 0.95
```

```
emmeans(by_country_model_age_exclusion, "Relationship_context", by="Sex")
```

```
## boundary (singular) fit: see ?isSingular
```

```
## Warning: Model failed to converge with 2 negative eigenvalues: -8.9e-06 -3.1e-05
```

```
## NOTE: Results may be misleading due to involvement in interactions
```

```
## Sex = -0.5:
##  Relationship_context emmean    SE    df lower.CL upper.CL
##                 -0.5  0.991 0.391  58.7    0.209    1.773
##                  0.5  1.343 0.387  56.0    0.568    2.119
##
## Sex =  0.5:
##  Relationship_context emmean    SE    df lower.CL upper.CL
##                 -0.5 -1.311 0.584 312.9   -2.460   -0.163
##                  0.5 -1.448 0.582 311.9   -2.593   -0.303
##
## Degrees-of-freedom method: kenward-roger
## Confidence level used: 0.95
```

```
emmeans(by_country_model_age_exclusion, "height_z_scored", by="Sex")
```

```
## boundary (singular) fit: see ?isSingular
```

```
## Warning: Model failed to converge with 2 negative eigenvalues: -8.9e-06 -3.1e-05
```

```
## NOTE: Results may be misleading due to involvement in interactions
```

```
## Sex = -0.5:
##  height_z_scored emmean    SE    df lower.CL upper.CL
##          -0.0178   1.17 0.353  37.2    0.451    1.883
##
## Sex =  0.5:
##  height_z_scored emmean    SE    df lower.CL upper.CL
##          -0.0178  -1.38 0.524 209.2   -2.413   -0.346
##
## Results are averaged over the levels of: Relationship_context
## Degrees-of-freedom method: kenward-roger
## Confidence level used: 0.95
```

```
emmeans(by_country_model_age_exclusion, "height_z_scored", by="Relationship_context")
```

```
## boundary (singular) fit: see ?isSingular
```

```
## Warning: Model failed to converge with 2 negative eigenvalues: -8.9e-06 -3.1e-05
```

```
## NOTE: Results may be misleading due to involvement in interactions
```

```
## Relationship_context = -0.5:
##  height_z_scored  emmean    SE   df lower.CL upper.CL
##          -0.0178 -0.1603 0.356 60.3   -0.873    0.552
##
## Relationship_context =  0.5:
##  height_z_scored  emmean    SE   df lower.CL upper.CL
##          -0.0178 -0.0522 0.354 59.4   -0.761    0.657
##
## Results are averaged over the levels of: Sex
## Degrees-of-freedom method: kenward-roger
## Confidence level used: 0.95
```

Model 2. Nationality Model

============================

```
data <- data %>%
    group_by(Nationality_selfreport, Sex) %>%
    mutate(
      height_preference = HEIGHTpref_CM - mean(HEIGHTpref_CM)
        ) %>%
    ungroup()
```

Model 2. Nationality Model No exclusions

```
nationality_model = lmer(height_preference ~ height_z_scored * Sex * Relationship_context + (1 + height_z_scored
 * Sex * Relationship_context || ID_NUMBER:Nationality_selfreport:Country) + (1 | Age), data=data, REML=FALSE)
```

```
## boundary (singular) fit: see ?isSingular
```

```
## Warning: Model failed to converge with 1 negative eigenvalue: -1.2e-01
```

```
summ(nationality_model)
```

```
## Warning in summ.merMod(nationality_model): Could not calculate r-squared. Try removing missing data
## before fitting the model.
```

**Observations**	1008
**Dependent variable**	height_preference

**Type**	Mixed effects linear regression

**AIC**	5972.10
**BIC**	6060.58

Fixed Effects

	Est.	S.E.	t val.	d.f.	p
**(Intercept)**	-0.02	0.28	-0.07	441.25	0.94
**height_z_scored**	1.65	0.28	5.84	224.66	0.00
**Sex**	-2.03	0.56	-3.62	441.25	0.00
**Relationship_context**	0.11	0.29	0.38	460.80	0.71
**height_z_scored:Sex**	-0.81	0.56	-1.44	224.66	0.15
**height_z_scored:Relationship_context**	0.74	0.29	2.59	227.73	0.01
**Sex:Relationship_context**	-0.55	0.59	-0.94	460.80	0.35
**height_z_scored:Sex:Relationship_context**	0.67	0.57	1.17	227.73	0.24

p values calculated using Satterthwaite d.f.

Random Effects

Group	Parameter	Std. Dev.
ID_NUMBER.Nationality_selfreport.Country	(Intercept)	3.97
ID_NUMBER.Nationality_selfreport.Country.1	height_z_scored	1.25
ID_NUMBER.Nationality_selfreport.Country.2	Sex	0.00
ID_NUMBER.Nationality_selfreport.Country.3	Relationship_context	2.43
ID_NUMBER.Nationality_selfreport.Country.4	height_z_scored:Sex	0.35
ID_NUMBER.Nationality_selfreport.Country.5	height_z_scored:Relationship_context	0.93
ID_NUMBER.Nationality_selfreport.Country.6	Sex:Relationship_context	2.83
ID_NUMBER.Nationality_selfreport.Country.7	height_z_scored:Sex:Relationship_context	0.40
Age	(Intercept)	0.00
Residual		2.54

Grouping Variables

Group	# groups	ICC
ID_NUMBER:Nationality_selfreport:Country	514	0.41
Age	50	0.04

Model 2. Nationality Model Both exclusions (sexual orientation and age)

```
nationality_model_both_exclusions = lmer(height_preference ~ height_z_scored * Sex * Relationship_context + (1 +
 height_z_scored * Sex * Relationship_context || ID_NUMBER:Nationality_selfreport:Country) + (1 | Age), data=subs
et(data, Age <=40 & Preferredsex_selfreport == 'PrefersOppositeSex'), REML=FALSE)
```

```
## boundary (singular) fit: see ?isSingular
```

```
## Warning: Model failed to converge with 3 negative eigenvalues: -1.9e-04 -3.4e-04
## -4.0e-04
```

```
summ(nationality_model_both_exclusions)
```

```
## Warning in summ.merMod(nationality_model_both_exclusions): Could not calculate r-squared. Try removing missing
data
## before fitting the model.
```

**Observations**	828
**Dependent variable**	height_preference
**Type**	Mixed effects linear regression

**AIC**	4780.38
**BIC**	4865.33

Fixed Effects

	Est.	S.E.	t val.	d.f.	p
**(Intercept)**	-0.01	0.27	-0.04	353.46	0.97

p values calculated using Satterthwaite d.f.

Fixed Effects

	Est.	S.E.	t val.	d.f.	p
**height_z_scored**	1.68	0.28	5.94	250.15	0.00
**Sex**	-2.93	0.54	-5.42	353.46	0.00
**Relationship_context**	0.18	0.31	0.58	346.93	0.56
**height_z_scored:Sex**	-0.84	0.56	-1.48	250.15	0.14
**height_z_scored:Relationship_context**	0.74	0.32	2.32	232.54	0.02
**Sex:Relationship_context**	-0.51	0.62	-0.83	346.93	0.41
**height_z_scored:Sex:Relationship_context**	0.73	0.64	1.15	232.54	0.25

p values calculated using Satterthwaite d.f.

Random Effects

Group	Parameter	Std. Dev.
ID_NUMBER.Nationality_selfreport.Country	(Intercept)	3.38
ID_NUMBER.Nationality_selfreport.Country.1	height_z_scored	1.18
ID_NUMBER.Nationality_selfreport.Country.2	Sex	0.00
ID_NUMBER.Nationality_selfreport.Country.3	Relationship_context	2.64
ID_NUMBER.Nationality_selfreport.Country.4	height_z_scored:Sex	1.41
ID_NUMBER.Nationality_selfreport.Country.5	height_z_scored:Relationship_context	1.21
ID_NUMBER.Nationality_selfreport.Country.6	Sex:Relationship_context	1.56
ID_NUMBER.Nationality_selfreport.Country.7	height_z_scored:Sex:Relationship_context	1.69
Age	(Intercept)	0.00
Residual		2.33

Grouping Variables

Group	# groups	ICC
ID_NUMBER:Nationality_selfreport:Country	422	0.34
Age	25	0.04

Model 2. Nationality Model sexual orientation but not age exclusion

```
nationality_model_sexual_orientation_exclusion = lmer(height_preference ~ height_z_scored * Sex * Relationship_co
ntext + (1 + height_z_scored * Sex * Relationship_context || ID_NUMBER:Nationality_selfreport:Country) + (1 | Ag
e), data=subset(data, Preferredsex_selfreport == 'PrefersOppositeSex'), REML=FALSE)
```

```
## boundary (singular) fit: see ?isSingular
```

```
## Warning: Model failed to converge with 5 negative eigenvalues: -2.2e-05 -5.1e-05
## -1.2e-04 -1.5e-04 -4.0e-04
```

```
summ(nationality_model_sexual_orientation_exclusion)
```

```
## Warning in summ.merMod(nationality_model_sexual_orientation_exclusion): Could not calculate r-squared. Try rem
oving missing data
## before fitting the model.
```

**Observations**	940
**Dependent variable**	height_preference
**Type**	Mixed effects linear regression

**AIC**	5442.47
**BIC**	5529.70

Fixed Effects

	Est.	S.E.	t val.	d.f.	p
**(Intercept)**	-0.00	0.26	-0.01	400.99	0.99
**height_z_scored**	1.69	0.27	6.29	282.19	0.00
**Sex**	-2.79	0.51	-5.43	400.99	0.00
**Relationship_context**	0.19	0.30	0.63	433.03	0.53
**height_z_scored:Sex**	-0.81	0.54	-1.51	282.19	0.13
**height_z_scored:Relationship_context**	0.75	0.29	2.60	167.74	0.01

p values calculated using Satterthwaite d.f.

Fixed Effects

	Est.	S.E.	t val.	d.f.	p
**Sex:Relationship_context**	-0.60	0.60	-1.00	433.03	0.32
**height_z_scored:Sex:Relationship_context**	0.56	0.58	0.97	167.74	0.33

p values calculated using Satterthwaite d.f.

Random Effects

Group	Parameter	Std. Dev.
ID_NUMBER.Nationality_selfreport.Country	(Intercept)	3.26
ID_NUMBER.Nationality_selfreport.Country.1	height_z_scored	1.54
ID_NUMBER.Nationality_selfreport.Country.2	Sex	0.03
ID_NUMBER.Nationality_selfreport.Country.3	Relationship_context	1.84
ID_NUMBER.Nationality_selfreport.Country.4	height_z_scored:Sex	0.29
ID_NUMBER.Nationality_selfreport.Country.5	height_z_scored:Relationship_context	0.27
ID_NUMBER.Nationality_selfreport.Country.6	Sex:Relationship_context	3.92
ID_NUMBER.Nationality_selfreport.Country.7	height_z_scored:Sex:Relationship_context	1.27
Age	(Intercept)	0.00
Residual		2.57

Grouping Variables

Group	# groups	ICC
ID_NUMBER:Nationality_selfreport:Country	479	0.27
Age	50	0.06

Model 2. Nationality Model age but not sexual orientation exclusion

```
nationality_model_age_exclusion = lmer(height_preference ~ height_z_scored * Sex * Relationship_context + (1 + he
ight_z_scored * Sex * Relationship_context || ID_NUMBER:Nationality_selfreport:Country) + (1 | Age), data=subset
(data, Age <=40), REML=FALSE)
```

```
## boundary (singular) fit: see ?isSingular
```

```
## Warning: Model failed to converge with 3 negative eigenvalues: -1.8e-05 -4.3e-05
## -1.5e-04
```

```
summ(nationality_model_age_exclusion)
```

```
## Warning in summ.merMod(nationality_model_age_exclusion): Could not calculate r-squared. Try removing missing d
ata
## before fitting the model.
```

**Observations**	892
**Dependent variable**	height_preference
**Type**	Mixed effects linear regression

**AIC**	5256.59
**BIC**	5342.88

Fixed Effects

	Est.	S.E.	t val.	d.f.	p
**(Intercept)**	-0.00	0.29	-0.02	396.43	0.99
**height_z_scored**	1.58	0.29	5.44	195.22	0.00
**Sex**	-2.13	0.58	-3.67	396.43	0.00
**Relationship_context**	0.12	0.30	0.40	375.80	0.69
**height_z_scored:Sex**	-0.72	0.58	-1.24	195.22	0.22
**height_z_scored:Relationship_context**	0.70	0.31	2.24	266.63	0.03
**Sex:Relationship_context**	-0.46	0.60	-0.78	375.80	0.44
**height_z_scored:Sex:Relationship_context**	0.81	0.63	1.29	266.63	0.20

p values calculated using Satterthwaite d.f.

Random Effects

Group	Parameter	Std. Dev.

Random Effects

Group	Parameter	Std. Dev.
ID_NUMBER.Nationality_selfreport.Country	(Intercept)	2.85
ID_NUMBER.Nationality_selfreport.Country.1	height_z_scored	0.59
ID_NUMBER.Nationality_selfreport.Country.2	Sex	5.56
ID_NUMBER.Nationality_selfreport.Country.3	Relationship_context	2.48
ID_NUMBER.Nationality_selfreport.Country.4	height_z_scored:Sex	1.46
ID_NUMBER.Nationality_selfreport.Country.5	height_z_scored:Relationship_context	1.37
ID_NUMBER.Nationality_selfreport.Country.6	Sex:Relationship_context	2.13
ID_NUMBER.Nationality_selfreport.Country.7	height_z_scored:Sex:Relationship_context	1.65
Age	(Intercept)	0.00
Residual		2.40

Grouping Variables

Group	# groups	ICC
ID_NUMBER:Nationality_selfreport:Country	455	0.13
Age	25	0.01

```
export_summs(nationality_model, nationality_model_both_exclusions, nationality_model_sexual_orientation_exclusio
n, nationality_model_age_exclusion, ci_level = 0.95, statistics = "all", error_format = "[{conf.low}, {conf.hig
h}]", model.names = list("Full Data", "Excluding based on sexual orientation and age", "Excluding based only on s
exual orientation", "Excluding based only on age"), to.file='Word', file.name='~/work/Papers/AssortativeHeight/Fr
ontierstable_model2_exclusion_comparison.docx')
```

```
## Warning in (function (model, scale = FALSE, confint = getOption("summ-confint", : Could not calculate r-square
d. Try removing missing data
## before fitting the model.
```

```
## Warning in (function (model, scale = FALSE, confint = getOption("summ-confint", : Could not calculate r-square
d. Try removing missing data
## before fitting the model.
```

```
## Warning in (function (model, scale = FALSE, confint = getOption("summ-confint", : Could not calculate r-square
d. Try removing missing data
## before fitting the model.
```

```
## Warning in (function (model, scale = FALSE, confint = getOption("summ-confint", : Could not calculate r-square
d. Try removing missing data
## before fitting the model.
```

```
## Warning in summ.merMod(model = new("lmerModLmerTest", vcov_varpar = structure(c(850.391997491431, : Could not
calculate r-squared. Try removing missing data
## before fitting the model.
```

```
## Warning in summ.merMod(model = new("lmerModLmerTest", vcov_varpar = structure(c(3.62292549112419, : Could not
calculate r-squared. Try removing missing data
## before fitting the model.
```

```
## Warning in summ.merMod(model = new("lmerModLmerTest", vcov_varpar = structure(c(0.00648778792303491, : Could n
ot calculate r-squared. Try removing missing data
## before fitting the model.
```

```
## Warning in summ.merMod(model = new("lmerModLmerTest", vcov_varpar = structure(c(12855.8005589168, : Could not
calculate r-squared. Try removing missing data
## before fitting the model.
```

	Full Data	Excluding based on sexual orientation and age	Excluding based only on sexual orientation	Excluding based only on age
(Intercept)	-0.02	-0.01	-0.00	-0.00
	[-0.57, 0.53]	[-0.54, 0.52]	[-0.51, 0.50]	[-0.57, 0.56]
height_z_scored	1.65 ***	1.68 ***	1.69 ***	1.58 ***
	[1.10, 2.20]	[1.12, 2.23]	[1.16, 2.22]	[1.01, 2.14]
Sex	-2.03 ***	-2.93 ***	-2.79 ***	-2.13 ***

	[-3.13, -0.93]	[-3.98, -1.87]	[-3.80, -1.78]	[-3.27, -0.99]
Relationship_context	0.11	0.18	0.19	0.12
	[-0.46, 0.68]	[-0.43, 0.78]	[-0.40, 0.78]	[-0.47, 0.70]
height_z_scored:Sex	-0.81	-0.84	-0.81	-0.72
	[-1.92, 0.29]	[-1.94, 0.27]	[-1.87, 0.24]	[-1.86, 0.41]
height_z_scored:Relationship_context	0.74 *	0.74 *	0.75 *	0.70 *
	[0.18, 1.31]	[0.12, 1.37]	[0.19, 1.32]	[0.09, 1.32]
Sex:Relationship_context	-0.55	-0.51	-0.60	-0.46
	[-1.70, 0.60]	[-1.72, 0.70]	[-1.78, 0.58]	[-1.63, 0.71]
height_z_scored:Sex:Relationship_context	0.67	0.73	0.56	0.81
	[-0.45, 1.80]	[-0.52, 1.99]	[-0.57, 1.70]	[-0.42, 2.03]
nobs	1008	828	940	892
sigma	2.54	2.33	2.57	2.40
logLik	-2968.05	-2372.19	-2703.23	-2610.30
AIC	5972.10	4780.38	5442.47	5256.59
BIC	6060.58	4865.33	5529.70	5342.88
deviance	5936.10	4744.38	5406.47	5220.59
df.residual	990.00	810.00	922.00	874.00
p.value				
r.squared				
r.squared.fixed				

group.nobs.ID_NUMBER:Nationality_selfreport:Country	514.00	422.00	479.00	455.00
group.nobs.Age	50.00	25.00	50.00	25.00

*** p < 0.001; ** p < 0.01; * p < 0.05.

Model 2. Nationality By Country model Broken down by Sex

Women Raters - No exclusions

```
nationality_model_women_raters = lmer(height_preference ~ height_z_scored * Relationship_context + (1 + height_z_
scored * Relationship_context || ID_NUMBER:Nationality_selfreport:Country) + (1 | Age), data=subset(data, Sex ==
 -0.5), REML=FALSE)
```

```
## Warning in checkConv(attr(opt, "derivs"), opt$par, ctrl = control$checkConv, :
## unable to evaluate scaled gradient
```

```
## Warning in checkConv(attr(opt, "derivs"), opt$par, ctrl = control$checkConv, :
## Model failed to converge: degenerate Hessian with 1 negative eigenvalues
```

```
## Warning: Model failed to converge with 1 negative eigenvalue: -8.5e-06
```

```
summ(nationality_model_women_raters)
```

```
## Warning in summ.merMod(nationality_model_women_raters): Could not calculate r-squared. Try removing missing da
ta
## before fitting the model.
```

**Observations**	633
**Dependent variable**	height_preference
**Type**	Mixed effects linear regression

**AIC**	3645.33
**BIC**	3689.84

Fixed Effects

Fixed Effects	Est.	S.E.	t val.	d.f.	p
(Intercept)	1.04	0.28	3.76	32.22	0.00
height_z_scored	2.21	0.36	6.14	157.13	0.00
Relationship_context	0.39	0.29	1.35	303.58	0.18
height_z_scored:Relationship_context	0.39	0.33	1.18	100.64	0.24

p values calculated using Satterthwaite d.f.

Random Effects

Group	Parameter	Std. Dev.
ID_NUMBER.Nationality_selfreport.Country	(Intercept)	3.47
ID_NUMBER.Nationality_selfreport.Country.1	height_z_scored	2.28
ID_NUMBER.Nationality_selfreport.Country.2	Relationship_context	2.82
ID_NUMBER.Nationality_selfreport.Country.3	height_z_scored:Relationship_context	0.76
Age	(Intercept)	0.18
Residual		2.18

Grouping Variables

Group	# groups	ICC
ID_NUMBER:Nationality_selfreport:Country	323	0.39
Age	43	0.17

Model 2. Women Raters Nationality Model Both exclusions (sexual orientation and age)

```
women_nationality_model_both_exclusions = lmer(height_preference ~ height_z_scored * Relationship_context + (1 +
 height_z_scored * Relationship_context || ID_NUMBER:Nationality_selfreport:Country) + (1 | Age), data=subset(dat
a,  Sex == -0.5 & Age <=40 & Preferredsex_selfreport == 'PrefersOppositeSex'), REML=FALSE)
```

```
## Warning in checkConv(attr(opt, "derivs"), opt$par, ctrl = control$checkConv, : Model is nearly unidentifiable:
large eigenvalue ratio
##  - Rescale variables?
```

```
## Warning: Model failed to converge with 1 negative eigenvalue: -1.5e-04
```

```
summ(women_nationality_model_both_exclusions)
```

```
## Warning in summ.merMod(women_nationality_model_both_exclusions): Could not calculate r-squared. Try removing m
issing data
## before fitting the model.
```

**Observations**	520
**Dependent variable**	height_preference
**Type**	Mixed effects linear regression

**AIC**	2865.47
**BIC**	2908.01

Fixed Effects

	Est.	S.E.	t val.	d.f.	p
**(Intercept)**	1.46	0.28	5.28	23.68	0.00
**height_z_scored**	2.15	0.33	6.56	142.29	0.00
**Relationship_context**	0.43	0.29	1.47	221.76	0.14
**height_z_scored:Relationship_context**	0.36	0.37	1.00	139.91	0.32

p values calculated using Satterthwaite d.f.

Random Effects

Group	Parameter	Std. Dev.
ID_NUMBER.Nationality_selfreport.Country	(Intercept)	2.97
ID_NUMBER.Nationality_selfreport.Country.1	height_z_scored	1.52
ID_NUMBER.Nationality_selfreport.Country.2	Relationship_context	2.16
ID_NUMBER.Nationality_selfreport.Country.3	height_z_scored:Relationship_context	1.67
Age	(Intercept)	0.29
Residual		2.07

Grouping Variables

Group	# groups	ICC
ID_NUMBER:Nationality_selfreport:Country	265	0.38
Age	23	0.10

Model 2. Women Raters Nationality Model sexual orientation but not age exclusion

```
women_nationality_model_sexual_orientation_exclusion = lmer(height_preference ~ height_z_scored * Relationship_co
ntext + (1 + height_z_scored * Relationship_context || ID_NUMBER:Nationality_selfreport:Country) + (1 | Age), dat
a=subset(data, Sex == -0.5 & Preferredsex_selfreport == 'PrefersOppositeSex'), REML=FALSE)
```

```
## Warning in checkConv(attr(opt, "derivs"), opt$par, ctrl = control$checkConv, :
## unable to evaluate scaled gradient
```

```
## Warning in checkConv(attr(opt, "derivs"), opt$par, ctrl = control$checkConv, :
## Model failed to converge: degenerate Hessian with 1 negative eigenvalues
```

```
## Warning: Model failed to converge with 1 negative eigenvalue: -1.1e-04
```

```
summ(women_nationality_model_sexual_orientation_exclusion)
```

```
## Warning in summ.merMod(women_nationality_model_sexual_orientation_exclusion): Could not calculate r-squared. T
ry removing missing data
## before fitting the model.
```

**Observations**	593
**Dependent variable**	height_preference
**Type**	Mixed effects linear regression

**AIC**	3307.73
**BIC**	3351.59

Fixed Effects

	Est.	S.E.	t val.	d.f.	p

p values calculated using Satterthwaite d.f.

Fixed Effects

	Est.	S.E.	t val.	d.f.	p
**(Intercept)**	1.39	0.26	5.44	34.16	0.00
**height_z_scored**	2.15	0.32	6.75	167.45	0.00
**Relationship_context**	0.49	0.29	1.70	277.83	0.09
**height_z_scored:Relationship_context**	0.47	0.34	1.38	113.99	0.17

p values calculated using Satterthwaite d.f.

Random Effects

Group	Parameter	Std. Dev.
ID_NUMBER.Nationality_selfreport.Country	(Intercept)	2.94
ID_NUMBER.Nationality_selfreport.Country.1	height_z_scored	1.74
ID_NUMBER.Nationality_selfreport.Country.2	Relationship_context	2.47
ID_NUMBER.Nationality_selfreport.Country.3	height_z_scored:Relationship_context	1.05
Age	(Intercept)	0.26
Residual		2.22

Grouping Variables

Group	# groups	ICC
ID_NUMBER:Nationality_selfreport:Country	302	0.36
Age	42	0.13

Model 2. Women Raters Nationality Model age but not sexual orientation exclusion

```
women_nationality_model_age_exclusion = lmer(height_preference ~ height_z_scored * Relationship_context + (1 + he
ight_z_scored * Relationship_context || ID_NUMBER:Nationality_selfreport:Country) + (1 | Age), data=subset(data,
 Sex == -0.5 & Age <=40), REML=FALSE)
```

```
## boundary (singular) fit: see ?isSingular
```

```
## Warning: Model failed to converge with 1 negative eigenvalue: -1.7e-04
```

```
summ(women_nationality_model_age_exclusion)
```

```
## Warning in summ.merMod(women_nationality_model_age_exclusion): Could not calculate r-squared. Try removing mis
sing data
## before fitting the model.
```

**Observations**	556
**Dependent variable**	height_preference
**Type**	Mixed effects linear regression

**AIC**	3150.49
**BIC**	3193.70

Fixed Effects

	Est.	S.E.	t val.	d.f.	p
**(Intercept)**	1.11	0.28	3.91	230.45	0.00
**height_z_scored**	2.05	0.35	5.82	130.22	0.00
**Relationship_context**	0.35	0.29	1.19	240.90	0.23
**height_z_scored:Relationship_context**	0.28	0.36	0.78	142.96	0.44

p values calculated using Satterthwaite d.f.

Random Effects

Group	Parameter	Std. Dev.
ID_NUMBER.Nationality_selfreport.Country	(Intercept)	3.54
ID_NUMBER.Nationality_selfreport.Country.1	height_z_scored	1.68
ID_NUMBER.Nationality_selfreport.Country.2	Relationship_context	2.81
ID_NUMBER.Nationality_selfreport.Country.3	height_z_scored:Relationship_context	1.57
Age	(Intercept)	0.00
Residual		1.82

Grouping Variables

Group Grouping Variables	# groups	ICC

Group	# groups	ICC
ID_NUMBER:Nationality_selfreport:Country	284	0.43
Age	24	0.10

```
export_summs(nationality_model_women_raters, women_nationality_model_both_exclusions, women_nationality_model_sex
ual_orientation_exclusion, women_nationality_model_age_exclusion, ci_level = 0.95, statistics = "all", digits=3,
 error_format = "[{conf.low}, {conf.high}]", model.names = list("Full Data", "Excluding based on sexual orientati
on and age", "Excluding based only on sexual orientation", "Excluding based only on age"), to.file='Word', file.n
ame='~/work/Papers/AssortativeHeight/Frontierstable_model2_exclusion_comparison_women_raters.docx')
```

```
## Warning in (function (model, scale = FALSE, confint = getOption("summ-confint", : Could not calculate r-square
d. Try removing missing data
## before fitting the model.
```

```
## Warning in (function (model, scale = FALSE, confint = getOption("summ-confint", : Could not calculate r-square
d. Try removing missing data
## before fitting the model.
```

```
## Warning in (function (model, scale = FALSE, confint = getOption("summ-confint", : Could not calculate r-square
d. Try removing missing data
## before fitting the model.
```

```
## Warning in (function (model, scale = FALSE, confint = getOption("summ-confint", : Could not calculate r-square
d. Try removing missing data
## before fitting the model.
```

```
## Warning in summ.merMod(model = new("lmerModLmerTest", vcov_varpar = structure(c(0.0172952698883274, : Could no
t calculate r-squared. Try removing missing data
## before fitting the model.
```

```
## Warning in summ.merMod(model = new("lmerModLmerTest", vcov_varpar = structure(c(0.0119617819799243, : Could no
t calculate r-squared. Try removing missing data
## before fitting the model.
```

```
## Warning in summ.merMod(model = new("lmerModLmerTest", vcov_varpar = structure(c(0.00992505992589222, : Could n
ot calculate r-squared. Try removing missing data
## before fitting the model.
```

```
## Warning in summ.merMod(model = new("lmerModLmerTest", vcov_varpar = structure(c(0.0193688166318116, : Could no
t calculate r-squared. Try removing missing data
## before fitting the model.
```

	Full Data	Excluding based on sexual orientation and age	Excluding based only on sexual orientation	Excluding based only on age
(Intercept)	1.041 ***	1.460 ***	1.393 ***	1.106 ***
	[0.498, 1.584]	[0.918, 2.002]	[0.891, 1.895]	[0.551, 1.661]
height_z_scored	2.207 ***	2.152 ***	2.146 ***	2.049 ***
	[1.502, 2.912]	[1.509, 2.795]	[1.523, 2.770]	[1.359, 2.739]
Relationship_context	0.386	0.427	0.491	0.345
	[-0.176, 0.947]	[-0.143, 0.998]	[-0.074, 1.056]	[-0.222, 0.913]
height_z_scored:Relationship_context	0.390	0.364	0.467	0.277
	[-0.256, 1.035]	[-0.352, 1.080]	[-0.198, 1.133]	[-0.421, 0.975]
nobs	633	520	593	556
sigma	2.178	2.066	2.222	1.818
logLik	-1812.667	-1422.734	-1643.867	-1565.247
AIC	3645.335	2865.468	3307.734	3150.495
BIC	3689.839	2908.007	3351.586	3193.702
deviance	3625.335	2845.468	3287.734	3130.495

df.residual	623.000	510.000	583.000	546.000
p.value				
r.squared				
r.squared.fixed				
group.nobs.ID_NUMBER:Nationality_selfreport:Country	323.000	265.000	302.000	284.000
group.nobs.Age	43.000	23.000	42.000	24.000

*** p < 0.001; ** p < 0.01; * p < 0.05.

Men Raters no exclusions

```
nationality_model_men_raters = lmer(height_preference ~ height_z_scored * Relationship_context + (1 + height_z_sc
ored * Relationship_context || ID_NUMBER:Nationality_selfreport:Country)  + (1 | Age), data=subset(data, Sex ==
0.5), REML=FALSE)
```

```
## boundary (singular) fit: see ?isSingular
```

```
## Warning: Model failed to converge with 1 negative eigenvalue: -7.6e-05
```

```
summ(nationality_model_men_raters)
```

```
## Warning in summ.merMod(nationality_model_men_raters): Could not calculate r-squared. Try removing missing data
## before fitting the model.
```

**Observations**	375
**Dependent variable**	height_preference
**Type**	Mixed effects linear regression

**AIC**	2309.80
**BIC**	2349.06

Fixed Effects

	Est.	S.E.	t val.	d.f.	p

p values calculated using Satterthwaite d.f.

Fixed Effects

	Est.	S.E.	t val.	d.f.	p
**(Intercept)**	-1.02	0.53	-1.93	190.15	0.06
**height_z_scored**	1.19	0.46	2.60	189.53	0.01
**Relationship_context**	-0.14	0.57	-0.24	186.25	0.81
**height_z_scored:Relationship_context**	1.08	0.49	2.19	185.64	0.03

p values calculated using Satterthwaite d.f.

Random Effects

Group	Parameter	Std. Dev.
ID_NUMBER.Nationality_selfreport.Country	(Intercept)	4.49
ID_NUMBER.Nationality_selfreport.Country.1	height_z_scored	0.00
ID_NUMBER.Nationality_selfreport.Country.2	Relationship_context	3.27
ID_NUMBER.Nationality_selfreport.Country.3	height_z_scored:Relationship_context	0.00
Age	(Intercept)	0.00
Residual		2.94

Grouping Variables

Group	# groups	ICC
ID_NUMBER:Nationality_selfreport:Country	191	0.51
Age	35	0.00

Model 2. Men Raters Nationality Model Both exclusions (sexual orientation and age)

```
men_nationality_model_both_exclusions = lmer(height_preference ~ height_z_scored * Relationship_context + (1 + he
ight_z_scored * Relationship_context || ID_NUMBER:Nationality_selfreport:Country) + (1 | Age), data=subset(data,
Sex == 0.5 & Age <=40 & Preferredsex_selfreport == 'PrefersOppositeSex'), REML=FALSE)
```

```
## boundary (singular) fit: see ?isSingular
```

```
summ(men_nationality_model_both_exclusions)
```

```
## Warning in summ.merMod(men_nationality_model_both_exclusions): Could not calculate r-squared. Try removing mis
sing data
## before fitting the model.
```

**Observations**	308
**Dependent variable**	height_preference
**Type**	Mixed effects linear regression

**AIC**	1884.18
**BIC**	1921.48

Fixed Effects

	Est.	S.E.	t val.	d.f.	p
**(Intercept)**	-1.41	0.53	-2.63	155.54	0.01
**height_z_scored**	1.14	0.48	2.39	155.28	0.02
**Relationship_context**	-0.03	0.63	-0.05	152.58	0.96
**height_z_scored:Relationship_context**	1.12	0.56	2.00	152.33	0.05

p values calculated using Satterthwaite d.f.

Random Effects

Group	Parameter	Std. Dev.
ID_NUMBER.Nationality_selfreport.Country	(Intercept)	4.15
ID_NUMBER.Nationality_selfreport.Country.1	height_z_scored	0.00
ID_NUMBER.Nationality_selfreport.Country.2	Relationship_context	3.62
ID_NUMBER.Nationality_selfreport.Country.3	height_z_scored:Relationship_context	0.00
Age	(Intercept)	0.00
Residual		2.82

Grouping Variables

Group	# groups	ICC
ID_NUMBER:Nationality_selfreport:Country	157	0.45

Grouping Variables

Group	# groups	ICC
Age	23	0.00

Model 2. Men Raters Nationality Model sexual orientation but not age exclusion

```
men_nationality_model_sexual_orientation_exclusion = lmer(height_preference ~ height_z_scored * Relationship_cont
ext + (1 + height_z_scored * Relationship_context || ID_NUMBER:Nationality_selfreport:Country) + (1 | Age), data=
subset(data, Sex == 0.5 & Preferredsex_selfreport == 'PrefersOppositeSex'), REML=FALSE)
```

```
## boundary (singular) fit: see ?isSingular
```

```
summ(men_nationality_model_sexual_orientation_exclusion)
```

```
## Warning in summ.merMod(men_nationality_model_sexual_orientation_exclusion): Could not calculate r-squared. Try
removing missing data
## before fitting the model.
```

**Observations**	347
**Dependent variable**	height_preference
**Type**	Mixed effects linear regression

**AIC**	2112.07
**BIC**	2150.56

Fixed Effects

	Est.	S.E.	t val.	d.f.	p
**(Intercept)**	-1.36	0.51	-2.67	149.33	0.01
**height_z_scored**	1.20	0.44	2.70	100.15	0.01
**Relationship_context**	-0.09	0.59	-0.15	172.36	0.88
**height_z_scored:Relationship_context**	1.02	0.51	2.00	171.67	0.05

p values calculated using Satterthwaite d.f.

Random Effects

Random Effects

Group Parameter Std. Dev.

Group	Parameter	Std. Dev.
ID_NUMBER.Nationality_selfreport.Country	(Intercept)	4.17
ID_NUMBER.Nationality_selfreport.Country.1	height_z_scored	0.50
ID_NUMBER.Nationality_selfreport.Country.2	Relationship_context	3.80
ID_NUMBER.Nationality_selfreport.Country.3	height_z_scored:Relationship_context	0.00
Age	(Intercept)	0.00
Residual		2.60

Grouping Variables

Group	# groups	ICC
ID_NUMBER:Nationality_selfreport:Country	177	0.45
Age	35	0.01

Model 2. Men Raters Nationality Model age but not sexual orientation exclusion

```
men_nationality_model_age_exclusion = lmer(height_preference ~ height_z_scored * Relationship_context + (1 + heig
ht_z_scored * Relationship_context || ID_NUMBER:Nationality_selfreport:Country) + (1 | Age), data=subset(data, Se
x == 0.5 & Age <=40), REML=FALSE)
```

```
## boundary (singular) fit: see ?isSingular
```

```
## Warning: Model failed to converge with 1 negative eigenvalue: -5.1e-05
```

```
summ(men_nationality_model_age_exclusion)
```

```
## Warning in summ.merMod(men_nationality_model_age_exclusion): Could not calculate r-squared. Try removing missi
ng data
## before fitting the model.
```

**Observations**	336
**Dependent variable**	height_preference
**Type**	Mixed effects linear regression

**AIC**	2081.67
**BIC**	2119.84

Fixed Effects

	Est.	S.E.	t val.	d.f.	p
**(Intercept)**	-1.05	0.55	-1.89	169.85	0.06
**height_z_scored**	1.17	0.50	2.35	169.64	0.02
**Relationship_context**	-0.09	0.59	-0.16	141.12	0.87
**height_z_scored:Relationship_context**	1.17	0.54	2.15	116.43	0.03

p values calculated using Satterthwaite d.f.

Random Effects

Group	Parameter	Std. Dev.
ID_NUMBER.Nationality_selfreport.Country	(Intercept)	4.58
ID_NUMBER.Nationality_selfreport.Country.1	height_z_scored	0.00
ID_NUMBER.Nationality_selfreport.Country.2	Relationship_context	3.43
ID_NUMBER.Nationality_selfreport.Country.3	height_z_scored:Relationship_context	0.81
Age	(Intercept)	0.00
Residual		2.87

Grouping Variables

Group	# groups	ICC
ID_NUMBER:Nationality_selfreport:Country	171	0.50
Age	23	0.00

```
export_summs(nationality_model_men_raters, men_nationality_model_both_exclusions, men_nationality_model_sexual_or
ientation_exclusion, men_nationality_model_age_exclusion, ci_level = 0.95, statistics = "all", digits=3, error_fo
rmat = "[{conf.low}, {conf.high}]", model.names = list("Full Data", "Excluding based on sexual orientation and ag
e", "Excluding based only on sexual orientation", "Excluding based only on age"), to.file='Word', file.name='~/wo
rk/Papers/AssortativeHeight/Frontierstable_model2_exclusion_comparison_men_raters.docx')
```

```
## Warning in (function (model, scale = FALSE, confint = getOption("summ-confint", : Could not calculate r-square
d. Try removing missing data
## before fitting the model.
```

```
## Warning in (function (model, scale = FALSE, confint = getOption("summ-confint", : Could not calculate r-square
d. Try removing missing data
## before fitting the model.
```

```
## Warning in (function (model, scale = FALSE, confint = getOption("summ-confint", : Could not calculate r-square
d. Try removing missing data
## before fitting the model.
```

```
## Warning in (function (model, scale = FALSE, confint = getOption("summ-confint", : Could not calculate r-square
d. Try removing missing data
## before fitting the model.
```

```
## Warning in summ.merMod(model = new("lmerModLmerTest", vcov_varpar = structure(c(0.00789138402470126, : Could n
ot calculate r-squared. Try removing missing data
## before fitting the model.
```

```
## Warning in summ.merMod(model = new("lmerModLmerTest", vcov_varpar = structure(c(2478.88193890807, : Could not
calculate r-squared. Try removing missing data
## before fitting the model.
```

```
## Warning in summ.merMod(model = new("lmerModLmerTest", vcov_varpar = structure(c(45934.7840493078, : Could not
calculate r-squared. Try removing missing data
## before fitting the model.
```

```
## Warning in summ.merMod(model = new("lmerModLmerTest", vcov_varpar = structure(c(0.00975341507692568, : Could n
ot calculate r-squared. Try removing missing data
## before fitting the model.
```

	Full Data	Excluding based on sexual orientation and age	Excluding based only on sexual orientation	Excluding based only on age
(Intercept)	-1.015	-1.408 **	-1.355 **	-1.047

	[-2.046, 0.015]	[-2.456, -0.360]	[-2.351, -0.360]	[-2.133, 0.039]
height_z_scored	1.189 *	1.142 *	1.200 **	1.166 *
	[0.293, 2.086]	[0.205, 2.079]	[0.329, 2.070]	[0.192, 2.141]
Relationship_context	-0.138	-0.030	-0.088	-0.095
	[-1.247, 0.970]	[-1.259, 1.200]	[-1.244, 1.068]	[-1.260, 1.070]
height_z_scored:Relationship_context	1.076 *	1.120 *	1.016 *	1.165 *
	[0.115, 2.038]	[0.021, 2.218]	[0.019, 2.014]	[0.103, 2.228]
nobs	375	308	347	336
sigma	2.942	2.820	2.599	2.867
logLik	-1144.898	-932.088	-1046.034	-1030.836
AIC	2309.795	1884.176	2112.068	2081.672
BIC	2349.065	1921.477	2150.561	2119.843
deviance	2289.795	1864.176	2092.068	2061.672
df.residual	365.000	298.000	337.000	326.000
p.value				
r.squared				
r.squared.fixed				
group.nobs.ID_NUMBER:Nationality_selfreport:Country	191.000	157.000	177.000	171.000
group.nobs.Age	35.000	23.000	35.000	23.000

*** p < 0.001; ** p < 0.01; * p < 0.05.

```
export_summs(nationality_model, nationality_model_women_raters, nationality_model_men_raters, ci_level = 0.95, st
atistics = "all", digits=3, error_format = "[{conf.low}, {conf.high}]", to.file='Word', model.names = list("Women
and Men", "Women", "Men"), file.name='~/work/Papers/AssortativeHeight/Frontierstable_model2_compare_sexes.docx')
```

```
## Warning in (function (model, scale = FALSE, confint = getOption("summ-confint", : Could not calculate r-square
d. Try removing missing data
## before fitting the model.
```

```
## Warning in (function (model, scale = FALSE, confint = getOption("summ-confint", : Could not calculate r-square
d. Try removing missing data
## before fitting the model.
```

```
## Warning in (function (model, scale = FALSE, confint = getOption("summ-confint", : Could not calculate r-square
d. Try removing missing data
## before fitting the model.
```

```
## Warning in summ.merMod(model = new("lmerModLmerTest", vcov_varpar = structure(c(850.391997491431, : Could not
calculate r-squared. Try removing missing data
## before fitting the model.
```

```
## Warning in summ.merMod(model = new("lmerModLmerTest", vcov_varpar = structure(c(0.0172952698883274, : Could no
t calculate r-squared. Try removing missing data
## before fitting the model.
```

```
## Warning in summ.merMod(model = new("lmerModLmerTest", vcov_varpar = structure(c(0.00789138402470126, : Could n
ot calculate r-squared. Try removing missing data
## before fitting the model.
```

	Women and Men	Women	Men
(Intercept)	-0.020	1.041 ***	-1.015
	[-0.571, 0.530]	[0.498, 1.584]	[-2.046, 0.015]
height_z_scored	1.649 ***	2.207 ***	1.189 *
	[1.095, 2.202]	[1.502, 2.912]	[0.293, 2.086]
Sex	-2.033 ***		
	[-3.133, -0.932]		

Relationship_context	0.111	0.386	-0.138
	[-0.463, 0.685]	[-0.176, 0.947]	[-1.247, 0.970]
height_z_scored:Sex	-0.815		
	[-1.921, 0.291]		
height_z_scored:Relationship_context	0.743 *	0.390	1.076 *
	[0.180, 1.305]	[-0.256, 1.035]	[0.115, 2.038]
Sex:Relationship_context	-0.551		
	[-1.699, 0.597]		
height_z_scored:Sex:Relationship_context	0.675		
	[-0.451, 1.800]		
nobs	1008	633	375
sigma	2.536	2.178	2.942
logLik	-2968.051	-1812.667	-1144.898
AIC	5972.102	3645.335	2309.795
BIC	6060.585	3689.839	2349.065
deviance	5936.102	3625.335	2289.795
df.residual	990.000	623.000	365.000
p.value			
r.squared			
r.squared.fixed			
group.nobs.ID_NUMBER:Nationality_selfreport:Country	514.000	323.000	191.000
group.nobs.Age	50.000	43.000	35.000

*** p < 0.001; ** p < 0.01; * p < 0.05.

Estimated Marginal Means for Model 2.

Model 2. Nationality Model - No exclusions

```
emmeans(nationality_model, "Sex", by="Relationship_context")
```

```
## boundary (singular) fit: see ?isSingular
```

```
## Warning: Model failed to converge with 4 negative eigenvalues: -9.7e-05 -1.4e-04
## -4.5e-04 -7.8e-04
```

```
## NOTE: Results may be misleading due to involvement in interactions
```

```
## Relationship_context = -0.5:
##   Sex emmean    SE    df lower.CL upper.CL
##  -0.5  0.803 0.359  84.2   0.0884   1.5173
##   0.5 -0.954 0.546 363.0  -2.0284   0.1203
##
## Relationship_context =  0.5:
##   Sex emmean    SE    df lower.CL upper.CL
##  -0.5  1.189 0.356  80.4   0.4815   1.8971
##   0.5 -1.119 0.544 362.2  -2.1879  -0.0501
##
## Degrees-of-freedom method: kenward-roger
## Confidence level used: 0.95
```

```
emmeans(nationality_model, "Relationship_context", by="Sex")
```

```
## boundary (singular) fit: see ?isSingular
```

```
## Warning: Model failed to converge with 4 negative eigenvalues: -9.7e-05 -1.4e-04
## -4.5e-04 -7.8e-04
```

```
## NOTE: Results may be misleading due to involvement in interactions
```

```
## Sex = -0.5:
##  Relationship_context emmean    SE    df lower.CL upper.CL
##                  -0.5  0.803 0.359  84.2   0.0884   1.5173
##                   0.5  1.189 0.356  80.4   0.4815   1.8971
##
## Sex =  0.5:
##  Relationship_context emmean    SE    df lower.CL upper.CL
##                  -0.5 -0.954 0.546 363.0  -2.0284   0.1203
##                   0.5 -1.119 0.544 362.2  -2.1879  -0.0501
##
## Degrees-of-freedom method: kenward-roger
## Confidence level used: 0.95
```

```
emmeans(nationality_model, "height_z_scored", by="Sex")
```

```
## boundary (singular) fit: see ?isSingular
```

```
## Warning: Model failed to converge with 4 negative eigenvalues: -9.7e-05 -1.4e-04
## -4.5e-04 -7.8e-04
```

```
## NOTE: Results may be misleading due to involvement in interactions
```

```
## Sex = -0.5:
##  height_z_scored emmean    SE  df lower.CL upper.CL
##         1.38e-15  0.996 0.321  51    0.352   1.6400
##
## Sex =  0.5:
##  height_z_scored emmean    SE  df lower.CL upper.CL
##         1.38e-15 -1.037 0.485 233   -1.992  -0.0815
##
## Results are averaged over the levels of: Relationship_context
## Degrees-of-freedom method: kenward-roger
## Confidence level used: 0.95
```

```
emmeans(nationality_model, "height_z_scored", by="Relationship_context")
```

```
## boundary (singular) fit: see ?isSingular
```

```
## Warning: Model failed to converge with 4 negative eigenvalues: -9.7e-05 -1.4e-04
## -4.5e-04 -7.8e-04
```

```
## NOTE: Results may be misleading due to involvement in interactions
```

```
## Relationship_context = -0.5:
##  height_z_scored  emmean    SE   df lower.CL upper.CL
##         1.38e-15 -0.0756 0.332 78.7   -0.737    0.585
##
## Relationship_context =  0.5:
##  height_z_scored  emmean    SE   df lower.CL upper.CL
##         1.38e-15  0.0352 0.330 77.7   -0.622    0.692
##
## Results are averaged over the levels of: Sex
## Degrees-of-freedom method: kenward-roger
## Confidence level used: 0.95
```

Model 2. Nationality Model - Both exclusions

```
emmeans(nationality_model_both_exclusions, "Sex", by="Relationship_context")
```

```
## boundary (singular) fit: see ?isSingular
```

```
## Warning: Model failed to converge with 1 negative eigenvalue: -1.3e-04
```

```
## NOTE: Results may be misleading due to involvement in interactions
```

```
## Relationship_context = -0.5:
##   Sex emmean    SE    df lower.CL upper.CL
##  -0.5   1.17 0.354  62.5    0.464    1.879
##   0.5  -1.46 0.541 349.1   -2.522   -0.393
##
## Relationship_context =  0.5:
##   Sex emmean    SE    df lower.CL upper.CL
##  -0.5   1.59 0.350  58.8    0.894    2.294
##   0.5  -1.57 0.539 347.0   -2.632   -0.511
##
## Degrees-of-freedom method: kenward-roger
## Confidence level used: 0.95
```

```
emmeans(nationality_model_both_exclusions, "Relationship_context", by="Sex")
```

```
## boundary (singular) fit: see ?isSingular
```

```
## Warning: Model failed to converge with 1 negative eigenvalue: -1.3e-04
```

```
## NOTE: Results may be misleading due to involvement in interactions
```

```
## Sex = -0.5:
##  Relationship_context emmean    SE    df lower.CL upper.CL
##                  -0.5   1.17 0.354  62.5    0.464    1.879
##                   0.5   1.59 0.350  58.8    0.894    2.294
##
## Sex =  0.5:
##  Relationship_context emmean    SE    df lower.CL upper.CL
##                  -0.5  -1.46 0.541 349.1   -2.522   -0.393
##                   0.5  -1.57 0.539 347.0   -2.632   -0.511
##
## Degrees-of-freedom method: kenward-roger
## Confidence level used: 0.95
```

```
emmeans(nationality_model_both_exclusions, "height_z_scored", by="Sex")
```

```
## boundary (singular) fit: see ?isSingular
```

```
## Warning: Model failed to converge with 1 negative eigenvalue: -1.3e-04
```

```
## NOTE: Results may be misleading due to involvement in interactions
```

```
## Sex = -0.5:
##  height_z_scored emmean   SE    df lower.CL upper.CL
##          -0.0332   1.38 0.31  34.7    0.753    2.012
##
## Sex =  0.5:
##  height_z_scored emmean   SE    df lower.CL upper.CL
##          -0.0332  -1.51 0.47 210.1   -2.442   -0.588
##
## Results are averaged over the levels of: Relationship_context
## Degrees-of-freedom method: kenward-roger
## Confidence level used: 0.95
```

```
emmeans(nationality_model_both_exclusions, "height_z_scored", by="Relationship_context")
```

```
## boundary (singular) fit: see ?isSingular
```

```
## Warning: Model failed to converge with 1 negative eigenvalue: -1.3e-04
```

```
## NOTE: Results may be misleading due to involvement in interactions
```

```
## Relationship_context = -0.5:
##  height_z_scored  emmean    SE   df lower.CL upper.CL
##          -0.0332 -0.1429 0.326 73.3   -0.793    0.508
##
## Relationship_context =  0.5:
##  height_z_scored  emmean    SE   df lower.CL upper.CL
##          -0.0332  0.0111 0.324 72.0   -0.635    0.658
##
## Results are averaged over the levels of: Sex
## Degrees-of-freedom method: kenward-roger
## Confidence level used: 0.95
```

Model 2. Nationality Model - Sexual Orientation exclusions Estimated

```
emmeans(nationality_model_sexual_orientation_exclusion, "Sex", by="Relationship_context")
```

```
## boundary (singular) fit: see ?isSingular
```

```
## Warning: Model failed to converge with 2 negative eigenvalues: -1.9e-05 -1.3e-03
```

```
## NOTE: Results may be misleading due to involvement in interactions
```

```
## Relationship_context = -0.5:
##   Sex emmean    SE    df lower.CL upper.CL
##  -0.5   1.13 0.334  87.5    0.465    1.794
##   0.5  -1.35 0.517 394.1   -2.367   -0.335
##
## Relationship_context =  0.5:
##   Sex emmean    SE    df lower.CL upper.CL
##  -0.5   1.62 0.330  82.4    0.959    2.273
##   0.5  -1.47 0.514 392.4   -2.481   -0.461
##
## Degrees-of-freedom method: kenward-roger
## Confidence level used: 0.95
```

```
emmeans(nationality_model_sexual_orientation_exclusion, "Relationship_context", by="Sex")
```

```
## boundary (singular) fit: see ?isSingular
```

```
## Warning: Model failed to converge with 2 negative eigenvalues: -1.9e-05 -1.3e-03
```

```
## NOTE: Results may be misleading due to involvement in interactions
```

```
## Sex = -0.5:
##  Relationship_context emmean    SE    df lower.CL upper.CL
##                  -0.5   1.13 0.334  87.5    0.465    1.794
##                   0.5   1.62 0.330  82.4    0.959    2.273
##
## Sex =  0.5:
##  Relationship_context emmean    SE    df lower.CL upper.CL
##                  -0.5  -1.35 0.517 394.1   -2.367   -0.335
##                   0.5  -1.47 0.514 392.4   -2.481   -0.461
##
## Degrees-of-freedom method: kenward-roger
## Confidence level used: 0.95
```

```
emmeans(nationality_model_sexual_orientation_exclusion, "height_z_scored", by="Sex")
```

```
## boundary (singular) fit: see ?isSingular
```

```
## Warning: Model failed to converge with 2 negative eigenvalues: -1.9e-05 -1.3e-03
```

```
## NOTE: Results may be misleading due to involvement in interactions
```

```
## Sex = -0.5:
##  height_z_scored emmean    SE    df lower.CL upper.CL
##         -0.00914   1.37 0.290  47.2    0.789    1.957
##
## Sex =  0.5:
##  height_z_scored emmean    SE    df lower.CL upper.CL
##         -0.00914  -1.41 0.446 231.7   -2.289   -0.532
##
## Results are averaged over the levels of: Relationship_context
## Degrees-of-freedom method: kenward-roger
## Confidence level used: 0.95
```

```
emmeans(nationality_model_sexual_orientation_exclusion, "height_z_scored", by="Relationship_context")
```

```
## boundary (singular) fit: see ?isSingular
```

```
## Warning: Model failed to converge with 2 negative eigenvalues: -1.9e-05 -1.3e-03
```

```
## NOTE: Results may be misleading due to involvement in interactions
```

```
## Relationship_context = -0.5:
##  height_z_scored  emmean    SE   df lower.CL upper.CL
##         -0.00914 -0.1107 0.311 92.7   -0.729    0.508
##
## Relationship_context =  0.5:
##  height_z_scored  emmean    SE   df lower.CL upper.CL
##         -0.00914  0.0724 0.309 91.3   -0.541    0.686
##
## Results are averaged over the levels of: Sex
## Degrees-of-freedom method: kenward-roger
## Confidence level used: 0.95
```

Model 2. Nationality Model - Age exclusions Estimated marginal means and

```
emmeans(nationality_model_age_exclusion, "Sex", by="Relationship_context")
```

```
## boundary (singular) fit: see ?isSingular
```

```
## Warning: Model failed to converge with 2 negative eigenvalues: -2.1e-04 -5.5e-04
```

```
## NOTE: Results may be misleading due to involvement in interactions
```

```
## Relationship_context = -0.5:
##   Sex emmean    SE    df lower.CL upper.CL
##  -0.5  0.853 0.377  62.0   0.0992   1.6061
##   0.5 -1.025 0.562 327.2  -2.1311   0.0809
##
## Relationship_context =  0.5:
##   Sex emmean    SE    df lower.CL upper.CL
##  -0.5  1.199 0.373  59.1   0.4516   1.9460
##   0.5 -1.158 0.560 326.1  -2.2602  -0.0554
##
## Degrees-of-freedom method: kenward-roger
## Confidence level used: 0.95
```

```
emmeans(nationality_model_age_exclusion, "Relationship_context", by="Sex")
```

```
## boundary (singular) fit: see ?isSingular
```

```
## Warning: Model failed to converge with 2 negative eigenvalues: -2.1e-04 -5.5e-04
```

```
## NOTE: Results may be misleading due to involvement in interactions
```

```
## Sex = -0.5:
##  Relationship_context emmean    SE    df lower.CL upper.CL
##                  -0.5  0.853 0.377  62.0   0.0992   1.6061
##                   0.5  1.199 0.373  59.1   0.4516   1.9460
##
## Sex =  0.5:
##  Relationship_context emmean    SE    df lower.CL upper.CL
##                  -0.5 -1.025 0.562 327.2  -2.1311   0.0809
##                   0.5 -1.158 0.560 326.1  -2.2602  -0.0554
##
## Degrees-of-freedom method: kenward-roger
## Confidence level used: 0.95
```

```
emmeans(nationality_model_age_exclusion, "height_z_scored", by="Sex")
```

```
## boundary (singular) fit: see ?isSingular
```

```
## Warning: Model failed to converge with 2 negative eigenvalues: -2.1e-04 -5.5e-04
```

```
## NOTE: Results may be misleading due to involvement in interactions
```

```
## Sex = -0.5:
##  height_z_scored emmean    SE  df lower.CL upper.CL
##          -0.0178   1.03 0.338  38    0.341    1.710
##
## Sex =  0.5:
##  height_z_scored emmean    SE  df lower.CL upper.CL
##          -0.0178  -1.09 0.500 212   -2.078   -0.105
##
## Results are averaged over the levels of: Relationship_context
## Degrees-of-freedom method: kenward-roger
## Confidence level used: 0.95
```

```
emmeans(nationality_model_age_exclusion, "height_z_scored", by="Relationship_context")
```

```
## boundary (singular) fit: see ?isSingular
```

```
## Warning: Model failed to converge with 2 negative eigenvalues: -2.1e-04 -5.5e-04
```

```
## NOTE: Results may be misleading due to involvement in interactions
```

```
## Relationship_context = -0.5:
##  height_z_scored  emmean    SE   df lower.CL upper.CL
##          -0.0178 -0.0862 0.343 63.3   -0.772    0.599
##
## Relationship_context =  0.5:
##  height_z_scored  emmean    SE   df lower.CL upper.CL
##          -0.0178  0.0205 0.341 62.3   -0.662    0.703
##
## Results are averaged over the levels of: Sex
## Degrees-of-freedom method: kenward-roger
## Confidence level used: 0.95
```
