# Supplementary material for: Assortative mate preferences for height across short-term and long-term relationship contexts in a cross-cultural sample
Source: Front Psychol. 2022 Aug 25;13:937146. doi: 10.3389/fpsyg.2022.937146 (PMC9454610; doi:10.3389/fpsyg.2022.937146)
Supplement: Supplementary file 3 [file Data_Sheet_3.DOCX]

Supplementary Material

# Supplementary Tables

**Table S1.** Open-ended responses of self-reported nationalities for full sample of raters (*N*=536).

| Nationality | *n* | Nationality | *n* | Nationality | *n* |
| --- | --- | --- | --- | --- | --- |
| Cuban | 186 | Spanish | 2 | Israelian | 1 |
| American | 106 | USA | 2 | Italian/French | 1 |
| Norwegian | 75 | Angolan | 1 | Italian/Dutch | 1 |
| East Asian | 72 | Armenia | 1 | Lithuanian | 1 |
| South Asian | 15 | British | 1 | North Asian | 1 |
| Middle Eastern | 14 | Canadian | 1 | Norwegian/ Swedish | 1 |
| White | 6 | Chinese | 1 | Somalian | 1 |
| Black | 5 | English | 1 | South American | 1 |
| Indian | 4 | Eritrea | 1 | South Korean | 1 |
| Polish | 4 | European | 1 | Sudan | 1 |
| Asian | 2 | Filipino | 1 | The former Yugoslav Republic of Macedonia | 1 |
| French | 2 | Finnish | 1 | Vietnamese | 1 |
| Italian | 2 | Icelandic | 1 | Unknown | 1 |

*Note: Nationalities are coded numerically in dataset.*

**Table S2.** Model 1 (full model with fixed and random effects): Linear mixed effects model testing for differences in assortative preferences for mate height as a function of a short-term versus long-term relationship context, nesting participants by country.

|  | Women and Men | Women only | Men only |
| --- | --- | --- | --- |
| (Intercept) | -0.103 | 1.167 *** | -1.244 * |
|  | [-0.678, 0.472] | [0.612, 1.722] | [-2.318, -0.169] |
| Own height (of rater, z-scored) | 1.892 *** | 2.461 *** | 1.453 ** |
|  | [1.304, 2.479] | [1.700, 3.222] | [0.519, 2.388] |
| Sex (of rater) | -2.374 *** |  |  |
|  | [-3.524, -1.224] |  |  |
| Relationship context | 0.112 | 0.392 | -0.140 |
|  | [-0.462, 0.686] | [-0.169, 0.953] | [-1.249, 0.969] |
| Own height * Sex | -0.681 |  |  |
|  | [-1.856, 0.494] |  |  |
| Own height * Relationship context | 0.735 * | 0.381 | 1.076 * |
|  | [0.172, 1.299] | [-0.265, 1.027] | [0.114, 2.038] |
| Sex * Relationship context | -0.558 |  |  |
|  | [-1.707, 0.590] |  |  |
| Own height * Sex * Relationship context | 0.690 |  |  |
|  | [-0.437, 1.816] |  |  |
| nobs | 1008 | 633 | 375 |
| sigma | 2.158 | 2.285 | 3.121 |
| logLik | -2992.948 | -1828.534 | -1152.944 |
| AIC | 6021.895 | 3677.068 | 2325.887 |
| BIC | 6110.378 | 3721.573 | 2365.157 |
| deviance | 5985.895 | 3657.068 | 2305.887 |
| df.residual | 990.000 | 623.000 | 365.000 |
| p.value |  |  |  |
| r.squared |  |  |  |
| r.squared.fixed |  |  |  |
| group.nobs.ID_NUMBER:Country | 514.000 | 323.000 | 191.000 |
| group.nobs.Age | 50.000 | 43.000 | 35.000 |
| Own height of rater is coded as “height_z_scored”; Sex of rater is coded as “Sex”; Relationship context has two levels: short-term, long-term; [Lower 95% CI, Upper 95% CI]; *** p < 0.001; ** p < 0.01; * p < 0.05. | | | |

**Table S3.** Model 1b (only male raters, inclusion/exclusion comparisons): Linear mixed effects model testing for differences in assortative preferences for mate height as a function of a short-term versus long-term relationship context, nesting participants by country.

|  | Full Data  (male raters only) | Excluding based on sexual orientation + age | Excluding based only on sexual orientation | Excluding based only on age |
| --- | --- | --- | --- | --- |
| (Intercept) | -1.244 * | -1.748 ** | -1.664 ** | -1.303 * |
|  | [-2.318, -0.169] | [-2.834, -0.662] | [-2.695, -0.633] | [-2.437, -0.168] |
| Own height (of rater, z-scored) | 1.453 ** | 1.421 ** | 1.475 ** | 1.443 ** |
|  | [0.519, 2.388] | [0.449, 2.392] | [0.572, 2.377] | [0.425, 2.461] |
| Relationship context | -0.140 | -0.033 | -0.089 | -0.098 |
|  | [-1.249, 0.969] | [-1.264, 1.197] | [-1.246, 1.067] | [-1.264, 1.067] |
| Own height * Relationship context | 1.076 * | 1.121 * | 1.016 * | 1.166 * |
|  | [0.114, 2.038] | [0.021, 2.220] | [0.018, 2.014] | [0.103, 2.229] |
| nobs | 375 | 308 | 347 | 336 |
| sigma | 3.121 | 2.965 | 3.219 | 3.558 |
| logLik | -1152.944 | -937.831 | -1052.312 | -1038.368 |
| AIC | 2325.887 | 1895.662 | 2124.624 | 2096.737 |
| BIC | 2365.157 | 1932.963 | 2163.117 | 2134.908 |
| deviance | 2305.887 | 1875.662 | 2104.624 | 2076.737 |
| df.residual | 365.000 | 298.000 | 337.000 | 326.000 |
| p.value |  |  |  |  |
| r.squared |  |  |  |  |
| r.squared.fixed |  |  |  |  |
| group.nobs.ID_NUMBER:Country | 191.000 | 157.000 | 177.000 | 171.000 |
| group.nobs.Age | 35.000 | 23.000 | 35.000 | 23.000 |
| Own height of rater is coded as “height_z_scored”; Relationship context has two levels: short-term, long-term. Excluding based on sexual orientation refers to models that include only participants who self-reported as preferring the opposite-sex. Excluding based on age refers to models that include only participants aged 15 to 40, as this may be considered the most reproductively relevant phase of the human lifespan.  [Lower 95% CI, Upper 95% CI]; *** p < 0.001; ** p < 0.01; * p < 0.05. | | | | |

**Table S4.** Model 1c (only female raters, inclusion/exclusion comparisons): Linear mixed effects model testing for differences in assortative preferences for mate height as a function of a short-term versus long-term relationship context, nesting participants by country.

|  | Full Data  (male raters only) | Excluding based on sexual orientation + age | Excluding based only on sexual orientation | Excluding based only on age |
| --- | --- | --- | --- | --- |
| (Intercept) | 1.167 *** | 1.629 *** | 1.478 *** | 1.284 *** |
|  | [0.612, 1.722] | [1.053, 2.205] | [0.944, 2.013] | [0.708, 1.859] |
| Own height (of rater, z-scored) | 2.461 *** | 2.340 *** | 2.304 *** | 2.331 *** |
|  | [1.700, 3.222] | [1.648, 3.032] | [1.634, 2.974] | [1.577, 3.085] |
| Relationship context | 0.392 | 0.417 | 0.481 | 0.353 |
|  | [-0.169, 0.953] | [-0.154, 0.987] | [-0.084, 1.046] | [-0.214, 0.920] |
| Own height * Relationship context | 0.381 | 0.329 | 0.438 | 0.262 |
|  | [-0.265, 1.027] | [-0.387, 1.045] | [-0.227, 1.104] | [-0.436, 0.960] |
| nobs | 633 | 520 | 593 | 556 |
| sigma | 2.285 | 2.236 | 2.356 | 2.255 |
| logLik | -1828.534 | -1432.172 | -1655.517 | -1580.823 |
| AIC | 3677.068 | 2884.344 | 3331.034 | 3181.647 |
| BIC | 3721.573 | 2926.882 | 3374.886 | 3224.855 |
| deviance | 3657.068 | 2864.344 | 3311.034 | 3161.647 |
| df.residual | 623.000 | 510.000 | 583.000 | 546.000 |
| p.value |  |  |  |  |
| r.squared |  |  |  |  |
| r.squared.fixed |  |  |  |  |
| group.nobs.ID_NUMBER:Country | 323.000 | 265.000 | 302.000 | 284.000 |
| group.nobs.Age | 43.000 | 23.000 | 42.000 | 24.000 |
| Own height of rater is coded as “height_z_scored”; Relationship context has two levels: short-term, long-term. Excluding based on sexual orientation refers to models that include only participants who self-reported as preferring the opposite-sex. Excluding based on age refers to models that include only participants aged 15 to 40, as this may be considered the most reproductively relevant phase of the human lifespan.  [Lower 95% CI, Upper 95% CI]; *** p < 0.001; ** p < 0.01; * p < 0.05. | | | | |

**Table S5.** Model 2 (full model with fixed and random effects): Linear mixed effects model testing for differences in assortative preferences for mate height as a function of a short-term versus long-term relationship context, nesting participants by self-reported nationality.

|  | Women and Men | Women only | Men only |
| --- | --- | --- | --- |
| (Intercept) | -0.020 | 1.041 *** | -1.015 |
|  | [-0.571, 0.530] | [0.498, 1.584] | [-2.046, 0.015] |
| Own height (of rater, z-scored) | 1.649 *** | 2.207 *** | 1.189 * |
|  | [1.095, 2.202] | [1.502, 2.912] | [0.293, 2.086] |
| Sex (of rater) | -2.033 *** |  |  |
|  | [-3.133, -0.932] |  |  |
| Relationship context | 0.111 | 0.386 | -0.138 |
|  | [-0.463, 0.685] | [-0.176, 0.947] | [-1.247, 0.970] |
| Own height * Sex | -0.815 |  |  |
|  | [-1.921, 0.291] |  |  |
| Own height * Relationship context | 0.743 * | 0.390 | 1.076 * |
|  | [0.180, 1.305] | [-0.256, 1.035] | [0.115, 2.038] |
| Sex * Relationship context | -0.551 |  |  |
|  | [-1.699, 0.597] |  |  |
| Own height * Sex * Relationship context | 0.675 |  |  |
|  | [-0.451, 1.800] |  |  |
| nobs | 1008 | 633 | 375 |
| sigma | 2.541 | 2.178 | 2.942 |
| logLik | -2968.051 | -1812.667 | -1144.898 |
| AIC | 5972.102 | 3645.335 | 2309.795 |
| BIC | 6060.585 | 3689.839 | 2349.065 |
| deviance | 5936.102 | 3625.335 | 2289.795 |
| df.residual | 990.000 | 623.000 | 365.000 |
| p.value |  |  |  |
| r.squared |  |  |  |
| r.squared.fixed |  |  |  |
| group.nobs.ID_NUMBER:Country | 514.000 | 323.000 | 191.000 |
| group.nobs.Age | 50.000 | 43.000 | 35.000 |
| Own height of rater is coded as “height_z_scored”; Sex of rater is coded as “Sex”; Relationship context has two levels: short-term, long-term; [Lower 95% CI, Upper 95% CI]; *** p < 0.001; ** p < 0.01; * p < 0.05. | | | |

**Table S6.** Model 2b (only male raters, inclusion/exclusion comparisons): Linear mixed effects model testing for differences in assortative preferences for mate height as a function of a short-term versus long-term relationship context, nesting participants by self-reported nationality..

|  | Full Data  (male raters only) | Excluding based on sexual orientation + age | Excluding based only on sexual orientation | Excluding based only on age |
| --- | --- | --- | --- | --- |
| (Intercept) | -1.015 | -1.408 ** | -1.355 ** | -1.047 |
|  | [-2.046, 0.015] | [-2.456, -0.360] | [-2.351, -0.360] | [-2.133, 0.039] |
| Own height (of rater, z-scored) | 1.189 * | 1.142 * | 1.200 ** | 1.166 * |
|  | [0.293, 2.086] | [0.205, 2.079] | [0.329, 2.070] | [0.192, 2.141] |
| Relationship context | -0.138 | -0.030 | -0.088 | -0.095 |
|  | [-1.247, 0.970] | [-1.259, 1.200] | [-1.244, 1.068] | [-1.260, 1.070] |
| Own height * Relationship context | 1.076 * | 1.120 * | 1.016 * | 1.165 * |
|  | [0.115, 2.038] | [0.021, 2.218] | [0.019, 2.014] | [0.103, 2.228] |
| nobs | 375 | 308 | 347 | 336 |
| sigma | 2.942 | 2.820 | 2.599 | 2.867 |
| logLik | -1144.898 | -932.088 | -1046.034 | -1030.836 |
| AIC | 2309.795 | 1884.176 | 2112.068 | 2081.672 |
| BIC | 2349.065 | 1921.477 | 2150.561 | 2119.843 |
| deviance | 2289.795 | 1864.176 | 2092.068 | 2061.672 |
| df.residual | 365.000 | 298.000 | 337.000 | 326.000 |
| p.value |  |  |  |  |
| r.squared |  |  |  |  |
| r.squared.fixed |  |  |  |  |
| group.nobs.ID_NUMBER:Country | 191.000 | 157.000 | 177.000 | 171.000 |
| group.nobs.Age | 35.000 | 23.000 | 35.000 | 23.000 |
| Own height of rater is coded as “height_z_scored”; Relationship context has two levels: short-term, long-term. Excluding based on sexual orientation refers to models that include only participants who self-reported as preferring the opposite-sex. Excluding based on age refers to models that include only participants aged 15 to 40, as this may be considered the most reproductively relevant phase of the human lifespan.  [Lower 95% CI, Upper 95% CI]; *** p < 0.001; ** p < 0.01; * p < 0.05. | | | | |

**Table S7.** Model 2c (only female raters, inclusion/exclusion comparisons): Linear mixed effects model testing for differences in assortative preferences for mate height as a function of a short-term versus long-term relationship context, nesting participants by self-reported nationality.

|  | Full Data  (male raters only) | Excluding based on sexual orientation + age | Excluding based only on sexual orientation | Excluding based only on age |
| --- | --- | --- | --- | --- |
| (Intercept) | 1.041 *** | 1.460 *** | 1.393 *** | 1.106 *** |
|  | [0.498, 1.584] | [0.918, 2.002] | [0.891, 1.895] | [0.551, 1.661] |
| Own height (of rater, z-scored) | 2.207 *** | 2.152 *** | 2.146 *** | 2.049 *** |
|  | [1.502, 2.912] | [1.509, 2.795] | [1.523, 2.770] | [1.359, 2.739] |
| Relationship context | 0.386 | 0.427 | 0.491 | 0.345 |
|  | [-0.176, 0.947] | [-0.143, 0.998] | [-0.074, 1.056] | [-0.222, 0.913] |
| Own height * Relationship context | 0.390 | 0.364 | 0.467 | 0.277 |
|  | [-0.256, 1.035] | [-0.352, 1.080] | [-0.198, 1.133] | [-0.421, 0.975] |
| nobs | 633 | 520 | 593 | 556 |
| sigma | 2.178 | 2.066 | 2.222 | 1.818 |
| logLik | -1812.667 | -1422.734 | -1643.867 | -1565.247 |
| AIC | 3645.335 | 2865.468 | 3307.734 | 3150.495 |
| BIC | 3689.839 | 2908.007 | 3351.586 | 3193.702 |
| deviance | 3625.335 | 2845.468 | 3287.734 | 3130.495 |
| df.residual | 623.000 | 510.000 | 583.000 | 546.000 |
| p.value |  |  |  |  |
| r.squared |  |  |  |  |
| r.squared.fixed |  |  |  |  |
| group.nobs.ID_NUMBER:Country | 323.000 | 265.000 | 302.000 | 284.000 |
| group.nobs.Age | 43.000 | 23.000 | 42.000 | 24.000 |
| Own height of rater is coded as “height_z_scored”; Relationship context has two levels: short-term, long-term. Excluding based on sexual orientation refers to models that include only participants who self-reported as preferring the opposite-sex. Excluding based on age refers to models that include only participants aged 15 to 40, as this may be considered the most reproductively relevant phase of the human lifespan.  [Lower 95% CI, Upper 95% CI]; *** p < 0.001; ** p < 0.01; * p < 0.05. | | | | |
